# Rhinoceros beetle (*Trypoxylus dichotomus*) cuticular hydrocarbons contain information about body size and sex

**DOI:** 10.1371/journal.pone.0299796

**Published:** 2024-03-14

**Authors:** Micah A. Bell, Garrett Lim, Chelsey Caldwell, Douglas J. Emlen, Brook O. Swanson

**Affiliations:** 1 Department of Biology, Gonzaga University, Spokane, Washington, United States of America; 2 Division of Biological Sciences, The University of Montana, Missoula, Montana, United States of America; Albert-Ludwigs-Universitat Freiburg, GERMANY

## Abstract

Japanese rhinoceros beetle (*Trypoxylus dichotomus*) males have exaggerated horns that are used to compete for territories. Larger males with larger horns tend to win these competitions, giving them access to females. Agonistic interactions include what appears to be assessment and often end without escalating to physical combat. However, it is unknown what information competitors use to assess each other. In many insect species chemical signals can carry a range of information, including social position, nutritional state, morphology, and sex. Specifically, cuticular hydrocarbons (CHCs), which are waxes excreted on the surface of insect exoskeletons, can communicate a variety of information. Here, we asked whether CHCs in rhinoceros beetles carry information about sex, body size, and condition that could be used by males during assessment behavior. Multivariate analysis of hydrocarbon composition revealed patterns associated with both sex and body size. We suggest that Rhinoceros beetles could be communicating information through CHCs that would explain behavioral decisions.

## Introduction

Long-chain hydrocarbons are secreted on the cuticle of most insects, referred to as cuticular hydrocarbons (CHCs). These are thought to have arisen in terrestrial arthropods to provide waterproofing and prevent desiccation [1]. However, it is currently understood that these non-volatile chemicals can also be used in communication, serving as indicators of several underlying physiological processes [1–3]. This wax layer is often composed of mixtures of long chain n-alkanes, methyl-branched alkanes, and alkenes [1]. Individual CHC profiles consist of a blend of different hydrocarbons, and the identities, concentrations, and ratios of these hydrocarbons differ widely both among and within species [1,3]. Here we test whether cuticular hydrocarbon profiles in the Japanese rhinoceros beetle contain the requisite information to be used as signals in social interactions.

Male Japanese rhinoceros beetles (*Trypoxylus dichotomus)* have large, sexually dimorphic horns that they use as weapons in contests with rival males over territories that include sap seeps used as a food resource [4–6]. Female beetles visit sap sites to feed, which provide territory holding males the opportunity to mate [4,5]. While some male-male interactions escalate to physical battle, where the horn is used as a tool to dislodge an opponent from the tree, many interactions result in one contestant retreating after an initial contact with the other competitor [5,7]. These aborted contests are indicative of some form of assessment, with smaller, poorer-condition males opting to end an encounter they are likely to lose prior to costly escalation (*reviewed in* [8]). However, Japanese rhinoceros beetles are nocturnal, and males have poor visual acuity [9], so it is not obvious how males might be assessing each other.

The length of the horn could function as a tactile signal, as in other beetles that battle in the dark (e.g., [10,11]), but observations of male contests suggest that males may also be able to determine critical information when they touch the cuticle of another beetle [5]. Insects are known to communicate characteristics such as sex, nutritional condition, and social dominance through chemical signals (*reviewed in* [2,12,13]. For instance in other beetles, males can identify females via contact with antennae [3,14,15]. Like other beetles, *Trypoxylus* has cuticular hydrocarbons on their surfaces and it is possible that interactions between beetles allow assessment through these chemicals.

CHC profiles often contain information about body size [2,16,17]. But CHC profiles can also change rapidly [13,18–21] and be costly to produce and maintain, making them signals of short-term body condition such as nutritional state [22]. If males can detect these compounds during agonistic encounters, then CHC profiles indicative of large body size and/or high body condition may deter physically disadvantaged males from suffering the costs of competition with larger, more aggressive males [1,16,23–26].

We examined the cuticular hydrocarbon composition of over 60 male and female beetles using gas chromatography-mass spectrometry. We hypothesized that there would be correlations between CHC profiles, sex, male body size (as measured by pronotum width), and male body condition (as estimated by mass relative to body size). If there are correlations between these variables and CHCs, then these chemicals could be used as information. This could help explain differences in observed male behavior when interacting with males and females, and it would help explain the apparent assessment behavior during male to male agonistic interactions.

## Materials and methods

### Husbandry and morphology

Beetle larvae were collected in the wild in Taiwan by a commercial supplier (LPS LLC, Denver, USA). Larvae were kept in individual containers and fed fermented hardwood sawdust [27]. Upon emergence from pupation, male beetles were kept in individual 5.7-L containers with pulped paper carton. Females were kept in groups of five per container. Beetles were kept in a 24˚ C lab space on a 14/10 light-dark cycle. Containers were sprayed with water daily to maintain humidity. Beetles were fed fresh apple slices every three days and had continuous access to water. Beetle morphological measurements were taken to the nearest 0.1 millimeter using digital calipers (Mitutoyo, Kawasaki, Japan). Head horn length (horn length) was measured in males as the straight-line distance from the clypeus to the longest horn tip [28,29]. Pronotum width was measured across the widest point of the pronotum and used as an indicator of overall body size. Beetle body mass was recorded weekly on an Ohaus Scout Pro balance (Parsippany, NJ, USA). The last mass taken before CHC analysis was used to estimate condition.

### CHC characterization and identification

Beetles were frozen before the extraction of CHCs. Only males without visible injuries or morphological abnormalities were used. The right elytron was used for analysis for all samples. Literature suggests CHCs vary in different body parts, but we chose the elytra as it contained the largest surface area for extraction [30,31]. After thawing, an elytron of a beetle was pulled outward away from the body. Using a Pasteur pipette, 1 mL of GC-MS grade hexanes (Fisher Chemical, Hampton, NH, USA) was dripped along the anterior end of the elytron and collected from the posterior end into a 2-mL autosampler screw-top glass vial. Samples were analyzed using gas chromatography-mass spectrometry (GC-MS) on an Agilent 7890B GC System fitted with an Agilent 5977B MS Detector (Agilent Technologies, Santa Clara, CA, USA). An Agilent J&W High-Resolution Gas Chromatography Column (HP-5ms (5%-phenyl)-methylpolysiloxane; 30m length, 0.250mm diameter, and 0.25μm film) was used. The GC was operated using the splitless mode with helium as the carrier gas. The method was programmed with an initial temperature of 50°C held for 1 minute and a ramp rate of 10°C per minute until reaching 300°C, which was held for 15 minutes resulting in a total run time of 41 minutes. Injection order and extraction orders were randomized across samples.

Samples were run in addition to a known standard of n-alkanes (Sigma-Aldrich standard solution C_21_- C_40_, St. Louis, MO, USA) and n-hexanes blanks every 5 samples to check for contamination. Mass spectrometry revealed the majority of peaks present in the CHC extraction samples all presented a major fragmenting ion of m/z = 57 which was also characteristic of the n-alkane standard. Using gas chromatogram spectra, peaks were labeled according to their retention time rounded to the nearest hundredth of a second. The area of each peak was found by integrating under the major ion of the compound using Agilent Mass Hunter (Agilent Technologies, Santa Clara, California). To account for variation in the total amount of CHCs extracted from each beetle, the ratio of individual peak areas to the total area of all peaks within a sample was used to determine the relative amounts of each compound on a beetle. CHC profiles were then created by listing the peaks found in a beetle’s CHC sample and associating the relative peak area value to each peak [32,33]. Kováts retention indices (RI) were calculated from interpolation between the retention times of the n-alkanes standard. Peak identifications should be considered hypotheses and although they are consistent with published data, it was not the focus of this paper to conduct exhaustive identification of compounds. Prediction of peak identities were made by comparing RIs and diagnostic ions to the MS data of identified CHCs in other studies and the NIST14 (National Institute of Standards & Technology, Gaithersburg, MD, USA) library database [34–38]. Double bond locations were not identified. Mass spectra of each peak are included in S1 Fig.

### Statistical analysis

CHC profiles were described using principal component (PC) analyses (JMP Pro 17 Cary, NC, USA). Two separate PC analyses were performed for two inquiry groups: One including both male and female samples (PC_All-beetles_) and one including only male samples (PC_Males_). CHCs were included in analyses if they occurred at > 0.1% mean abundance. ANOVA was used to ask whether there were differences in PC values between male and female samples. For the male-only samples, individual linear models were used to examine relationships between PCs of the hydrocarbon mixture and prothorax width, horn length, and male body condition. Additional linear models were used to test relationships between individual CHCs identified from the eigenvectors. Condition was estimated as the residual mass calculated from the empirical, linear relationship between pronotum width and mass.

## Results

### Sex

**Fig 1:**
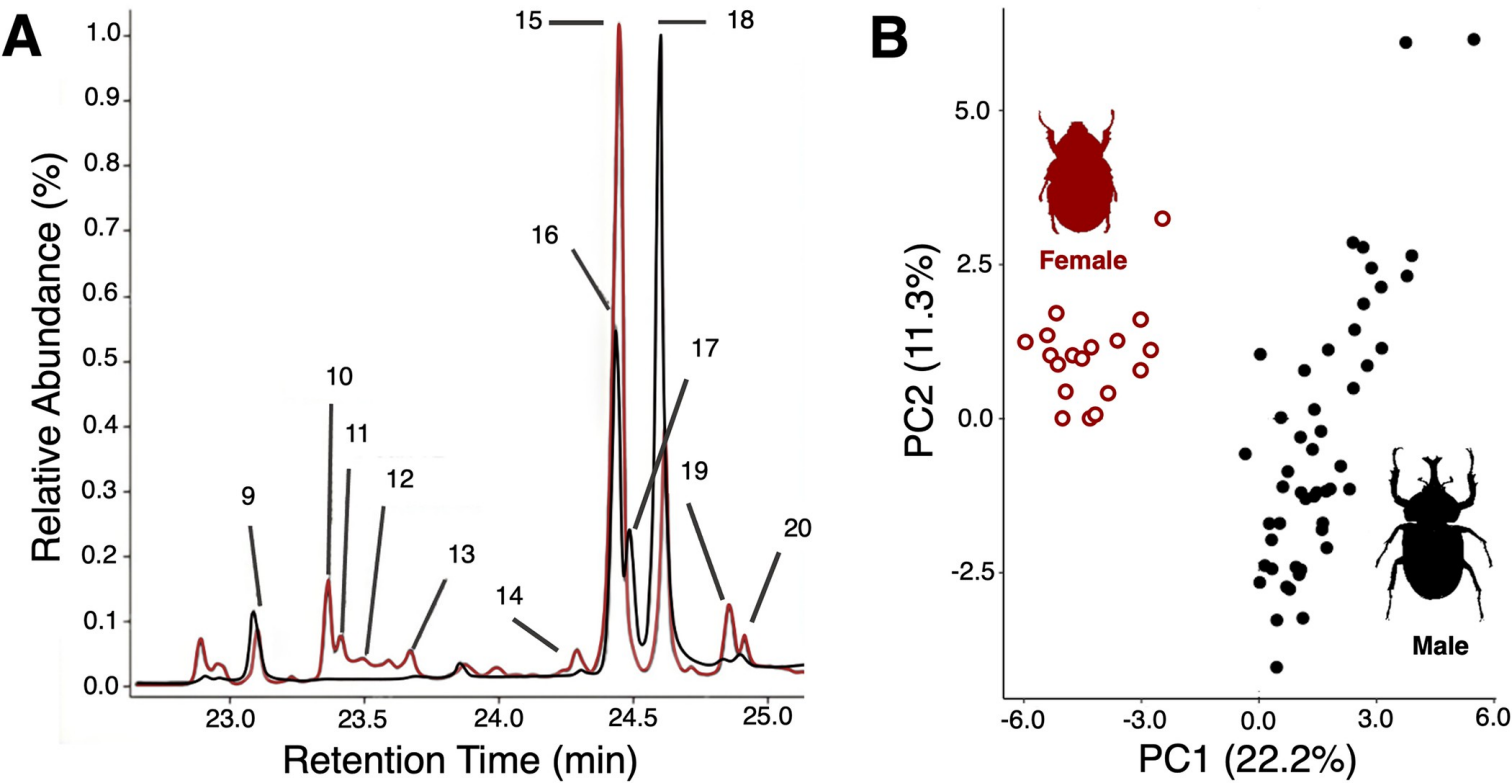
Male and female rhinoceros beetles (*Trypxoylus dichotomus*) vary in cuticular hydrocarbon profile. (A) Comparison of truncated gas chromatogram spectra obtained from samples made by washing the elytra of a male beetle (depicted in black) and female beetle (depicted in red) with hexanes. Peaks 11,12, and 15 are present across 18 female samples and were not found in any of the 49 males while peaks 16 and 17 are present in males and not females. (B) Principal component 1 (PC1_All-beetles_) vs. principal component 2 (PC2_All-beetles_) of the relative abundance of cuticular hydrocarbons found in both male (black solid markers) and female (red open markers) beetles.

37 unique hydrocarbons were identified across all 66 beetles. More hydrocarbons were present in males [39] above 0.1% mean abundance than females (25, see Table 1). CHC profiles from 18 females and 49 males were used to analyze cuticular hydrocarbon sex differences in the principal component analysis (PC_All-beetles_). The first principal component (PC1_All-beetles_) could explain greater than 20% of the variation in the data and was different between sexes (Table 2; F_(1,65)_ = 345.0499, p = <0.0001). PC2_All-beetles_ and PC3_All-beetles_ explained greater than 10% and 5% respectively. Eigenvectors revealed that PC1_All-beetles_ was mainly driven by low concentrations of peaks 11, 12, 15, and 20, and high concentrations of peaks 3, 16, and 17. Males score higher on PC1_All-beetles_ than females, and peaks 11, 12, and 15 were absent in males and peaks 16 and 17 were absent in females (Fig 1).

**Table 1:** List of likely cuticular hydrocarbons (CHCs) present on the elytra of male and female Japanese rhinoceros beetles (*Trypoxylus dichotomus)*.

Peak Number	RT (min)	RI	Tentative Structure	% Abundance Males (n = 49)		% Abundance Females (n = 17)	
				Mean	Std. Dev.	Mean	Std. Dev.
1	19.63	2100	Heneicosane	0.3	0.5	0.0	0.1
2	20.54	2200	Docosane	0.5	0.4	0.3	0.6
3	21.43	2300	Tricosane	4.8	2.1	1.5	0.9
4	22.23	2395	Unknown	0.1	0.2	0.2	0.5
5	22.27	2400	Tetracosane	1.0	0.9	0.2	0.3
6	22.80	2466	3-methyltetracosane	0.0	0.1	0.0	0.0
7	22.88	2476	Pentacosadiene*	1.1	1.1	2.2	1.6
8	22.96	2485	Pentacosene*	0.6	0.5	1.3	0.8
9	23.08	2500	Pentacosane	9.1	4.2	4.3	2.0
10	23.28	2526	Unknown	0.0	0.1	0.0	0.1
11	23.33	2532	Pentacosadiene*	0.0	0.0	5.6	1.8
12	23.46	2549	Pentacosadiene*	0.0	0.2	4.6	2.9
13	23.86	2600	Hexacosane	1.7	1.1	2.8	2.5
14	24.38	2672	3-methylhexacosane	0.5	0.5	0.3	0.3
15	24.43	2678	Heptacosadiene*	0.0	0.0	41.9	9.3
16	24.44	2680	Heptacosene*	29.3	11.2	0.0	0.0
17	24.47	2684	Heptacosene*	8.0	4.3	0.0	0.0
18	24.59	2700	Heptacosane	29.5	11.4	21.8	6.8
19	24.79	2728	Unknown	1.9	9.0	0.2	0.4
20	24.88	2741	Heptacosadiene*	1.1	0.9	4.7	1.3
21	24.98	2755	Heptacosadiene*	0.4	0.9	1.6	1.4
22	25.05	2764	Unknown	0.4	0.3	0.1	0.2
23	25.12	2774	3-methylheptacosane	0.1	0.2	0.0	0.0
24	25.31	2800	Octacosane	1.2	1.3	0.3	0.5
25	25.74	2862	4-methyloctacosane	0.0	0.0	0.0	0.0
26	25.77	2866	3-methyloctacosane	0.0	0.1	0.0	0.0
27	25.90	2884	Nonacosene*	2.7	2.1	0.7	0.5
28	26.01	2900	Nonacosane	4.3	4.1	2.5	1.3
29	26.18	2924	Nonacosadiene*	0.0	0.0	0.2	0.4
30	26.25	2934	Unknown	1.2	1.2	2.2	1.0
31	26.31	2943	5-methylnonacosane	0.0	0.1	0.1	0.2
32	26.43	2959	3-methylnonacosane	0.0	0.1	0.0	0.0
33	26.72	3000	Triacontane	0.7	1.1	0.1	0.2
34	27.52	3100	Hentriacontane	1.0	1.9	0.1	0.2
35	28.44	3200	Dotriacontane	0.3	0.4	0.0	0.0
36	29.51	3300	Tritriacontane	0.1	0.2	0.0	0.0
37	30.77	3400	Tetratriacontane	0.0	0.1	0.0	0.0

CHCs collected from the right elytron and analyzed through gas chromatography-mass spectrometry. Peak number corresponds to the order of elution from the column. RT = Retention time. RI = Kováts Rention Index. Tentative structure determined by comparison of RIs, diagnostic ions, C21-C40 n-alkanes standard, and the NIST14 library database. Mean percent abundance and standard deviation reported in male and female samples respectively.

* Double bond placement undetermined.

**Table 2:** Principal component (PC) information for the two PC analyses of cuticular hydrocarbon composition.

PCAll-beetles	Eigenvalue	Percent	Cumulative Percent	PCMales	Eigenvalue	Percent	Cumulative Percent
1	8.2182	22.211	22.211	1	6.4356	18.387	18.387
2	4.1814	11.301	33.512	2	4.0459	11.560	29.947
3	3.2152	8.690	42.202	3	3.1533	9.009	38.957
4	2.3997	6.486	48.688	4	2.6539	7.583	46.540
5	1.9637	5.307	53.995	5	1.8959	5.417	51.956
6	1.6814	4.544	58.540	6	1.7593	5.027	56.983
7	1.6134	4.360	62.900	7	1.6893	4.827	61.809
8	1.3207	3.570	66.470	8	1.4395	4.113	65.922
9	1.247	3.370	69.840	9	1.2633	3.610	69.532
10	1.1802	3.190	73.030	10	1.2127	3.465	72.997
11	1.0982	2.968	75.998	11	1.1178	3.194	76.190
12	1.0407	2.813	78.811	12	1.0931	3.123	79.313
13	0.9629	2.603	81.413	13	0.9578	2.736	82.050

PC_All-beetles_ is for male and female beetles and PC_Males_ is for male beetles only.

### Male body size

**Fig 2:**
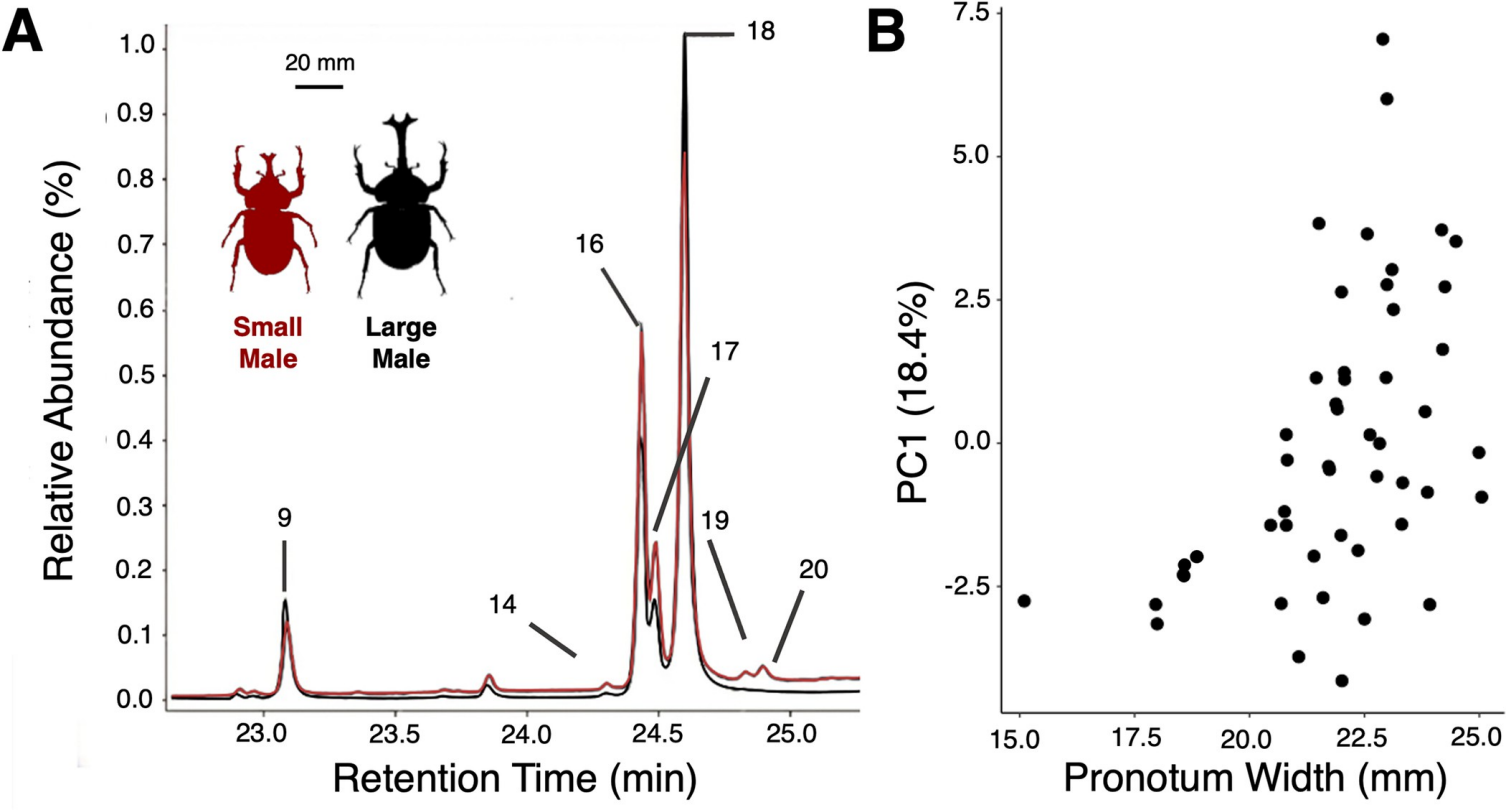
Different sized male Japanese rhinoceros beetles (*Trypxoylus dichotomus*) vary in cuticular hydrocarbon profile. (A) Comparison of truncated gas chromatogram spectra obtained from samples made by washing the elytron of a relatively large male beetle (black line; pronotum width 22.91mm) and smaller male beetle (red line; pronotum width 20.80mm) with hexanes. Males produce the same peaks across different body sizes, but vary in relative abundance. (B) Principal component 1 (PC1_Males_) of the relative abundance of cuticular hydrocarbons found in male beetles vs pronotum width. High scores on PC1 are associated with larger body size.

Nearly all males presented the same CHCs on their elytra but individuals varied in relative concentrations. Peaks 16–18 were consistently the highest concentrated hydrocarbons in all samples (Table 1). CHC profiles from 49 males were used to analyze CHC profiles in relation to body size in a principal component analysis. The first principal component (PC1_Males_) could explain >15% of the variation in the data (PC2_Males_ and PC3_Males_ explaining greater than 10% and 5% respectively; Table 2). Eigenvectors revealed that PC1_Males_ was mainly driven by high concentrations of peaks 18 and low concentrations of peaks 16 and 17. Larger males scored higher than smaller males. PC1_Males_ had a positive relationship with body size (Fig 2; F_1,48_ = 13.3761, *p* = 0.0006) and horn length (F_1,48_ = 9.3023, *p* = 0.0038). PC2_Males_ and PC3_Males_ did not correlate with body size (PC2_Males_: F_1,48_ = 0.0022, *p* = 0.9632; PC3_Males_: F_1,48_ = 3.1817, *p* = 0.0809).

**Fig 3:**
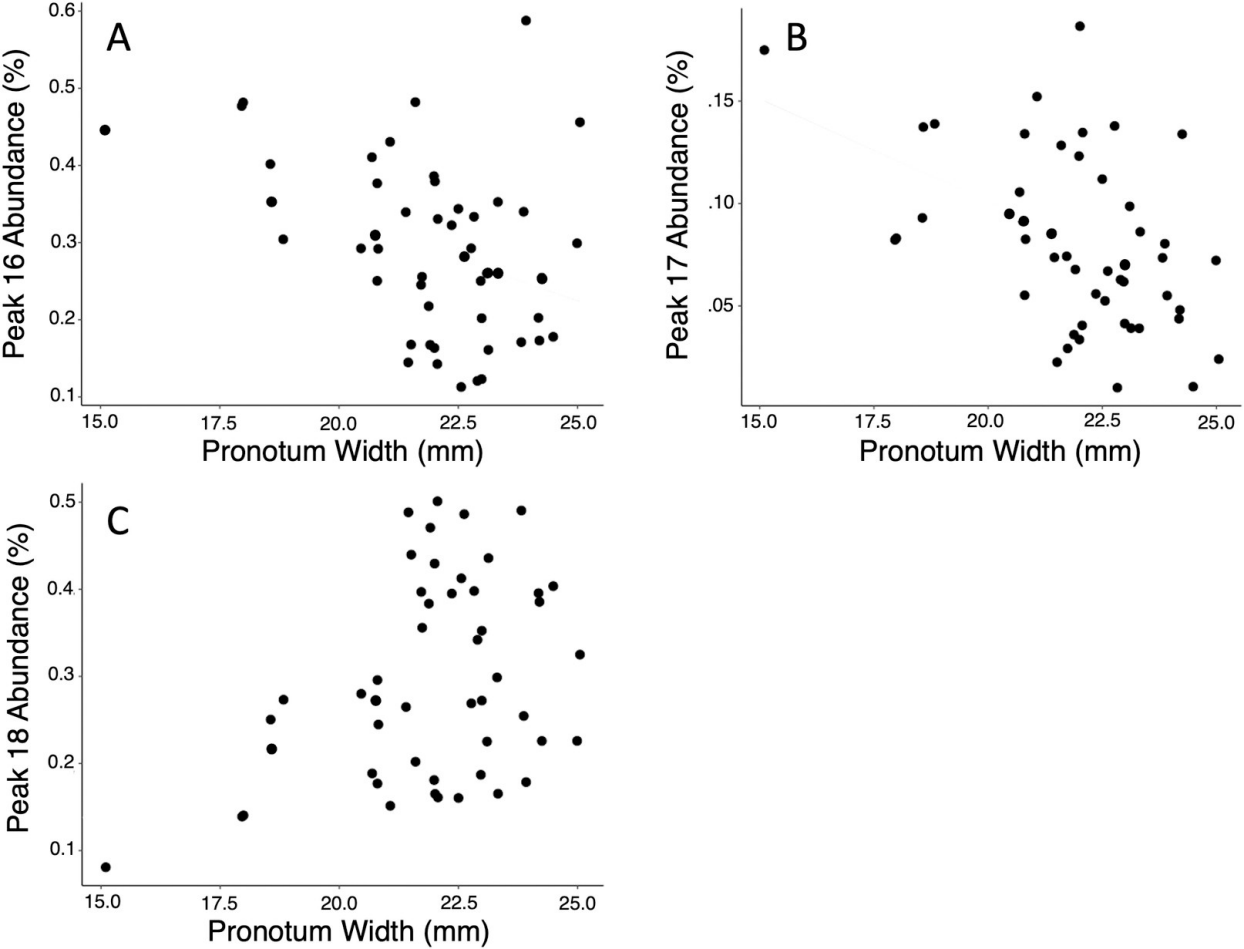
Scatter plot of the relative concentrations of peaks 16–18 against pronotum width of male Japanese rhinoceros beetles (*Trypoxylus dichotomus)*. Peaks 16 (A) and 17 (B) are both negatively associated with body size and peak 18 (C) is positively associated with body size.

The concentration of peak 18 had a positive relationship with body size (Fig 3; F_1,48_ = 8.0573, *p* = 0.0067) but not horn length (F_1,48_ = 1.615, *p* = 0.2867). The concentration of peaks 16 and 17 had negative relationships with body size (Fig 3; Peak 16: F_1,48_ = 11.1977, *p* = 0.0082; Peak 17: F_1,48_ = 13.1434, *p* = 0.0007). The concentration of peak 17 also had a negative relationship with horn size (F_1,48_ = 5.5667 *p* = 0.0225) but peak 16 did not (F_1,48_ = 2.3201, *p* = 0.1344).

### Male condition

**Fig 4:**
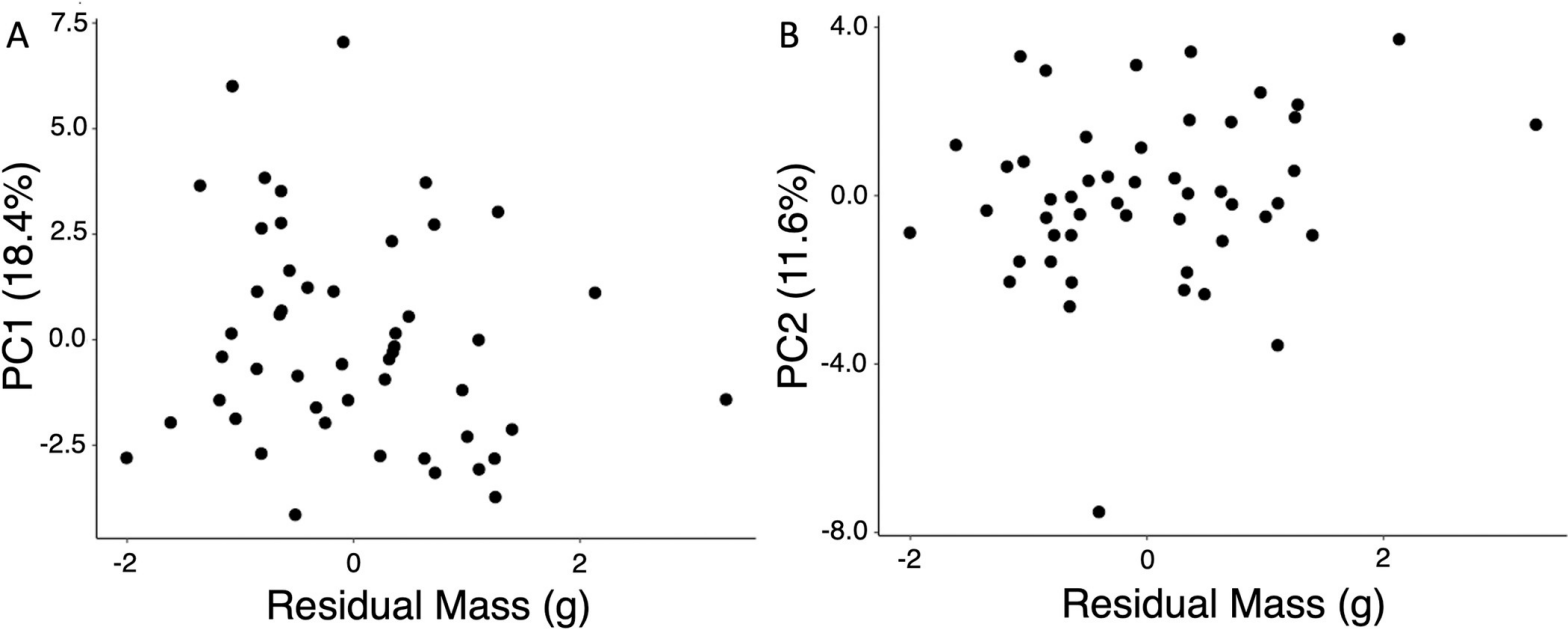
Scatter plots of PC1_Males_ (A) and PC2_Males_ (B) against condition. Condition was estimated by taking the residuals of the regression relationship between mass and pronotum width. There is no relationship between PC1_Males_ or PC2_Males_ and condition.

Condition was estimated as the mass deviation (residuals) from the empirical relationship between mass and prothorax width. This condition estimate describes how heavy an individual is compared to its exoskeleton size. The PC_(Males)_ analysis was used to compare condition with CHC profile. There was no observed relationship between either of the first two principal components and this estimate of condition (Fig 4; PC1_Males_: F_1,48_ = 1.4631, *p* = 0.2325; PC2_Males_: F_1,48_ = 2.0991, *p* = 0.1540). The measurement and CHC data for the individuals used in all of these analyses are included in Supporting File 1.

## Discussion

Information about sex and male body size, but not male short-term condition, is correlated with relative concentrations of CHCs in Japanese rhinoceros beetles. The abundance and presence/absence of particular hydrocarbons varied between the sexes (Table 1 and Fig 1). CHCs are known to be utilized in mate recognition and selection in other insects [16,18,40], and rhinoceros beetles may identify female conspecifics via the presence of peaks 11, 12 (both consistent with versions of pentacosadiene), and 15(a Heptacosadiene). Male sex identity was associated with the presence of peaks 16 and 17 (both consistent with heptacosenes) (Fig 1). These patterns could explain behavioral decisions made by male beetles. When encountering a conspecific, males will either initiate courtship or assume an aggressive posture [5,7]. The differences in CHCs between males and females could explain this behavioral dichotomy, allowing males to quickly identify the sex of a conspecific on a territory and initiate an appropriate behavioral response [3,14,15,41]. For instance, in another species of beetle, the presence of specific Methylpentacosanes on females is required to elicit mating behavior in males [14]. Additionally, in long-horned beetles, a mixture of monounsaturated hydrocarbons on a centrifuge tube is sufficient to elicit mating behavior from males [15]. Our data are consistent with similar functions. However, whether these hydrocarbons contain information about female characteristics and play a role in male mate selection is unknown. Clearly, CHCs will have to be manipulated to test whether these compounds produce sex-specific behavior in *Trypxoylus* [1,12].

Furthermore, during agonistic interactions males sometimes fight, but more commonly one competitor retreats [4,5,7]. This is similar to many other species where ritualized assessment should prevent injury to weaker competitors [42–45]. Male beetles may be using the exaggerated horn as a signal of competitive ability, however beetles seem to have poor vision and most competitive interactions take place in the dark [4,5,7,9]. We find that CHCs vary with body size and could therefore provide information to estimate the competitive ability of a rival (Figs 2 and 3). Therefore, CHCs could be a signal allowing Japanese rhinoceros beetles to assess the potential costs of escalating a fight. In dung beetles CHCs are also different across male size and morphotype. This variation could be a signal to other conspecifics or could be due to different desiccation risks for the different morphs [22]. In flour beetles, the amount of CHCs produced (which was not measured in our study) is correlated with both body size and fighting ability and seems to be under directional selection. However, CHC composition (as measured in our study) is assessed by females and is under selection through mate choice [16]. Although we do not know if females are actively making mating choices in *Trypxoylus*, male CHCs could provide information to female beetles about the quality of males and could therefore be used in female mate choice [2,16,18,25].

In contrast to sex and size, we find no evidence that CHCs reflect short term condition (Fig 4). In some other systems CHCs can change quickly to reflect changes in condition, diet, or social hierarchy [22,24,25]. It is possible that the condition variation in our beetles was not large enough to affect CHCs. Hydration status can also affect beetle mass and this may mask true variation in physiological condition. However, all of the beetles in this study had continuous access to water throughout the experiment, suggesting that the mass deviations were not due to hydration alone. While the biosynthetic pathways of rhinoceros beetles were not explored, the structure of a CHC (chain length, saturation, and functional groups) is regulated through interactions between elongases, desaturases, and monomeric subunits used in biosynthesis [1]. In *Trypoxylus*, it is known that nutritional availability during the larval stage can lead to differences in mature male horn size, body proportionality, and wing size [27]. This trend is also present in stag beetles (*Cylommatus metalifer*), where larval nutrition levels affect mandible length [46]. It is possible that CHC biosynthesis pathways are similarly shaped by larval conditions during development [27].

In our analysis, CHCs were only extracted from the elytra. The possibility of CHC compositions varying in different regions of the body of rhinoceros beetles remains unexplored. In some insect species, CHCs differ among body parts, aiding in deciphering the position and orientation of conspecifics [30,31]. Future studies will be needed to determine whether CHCs extracted from other regions of the beetle’s body provide distinct CHC profiles and possibly different information.

The results presented here bring up a number of additional questions, and future research will attempt to clarify the function of hydrocarbons in beetle behavior. Although it appears that there is information available in these hydrocarbons, and there should be opportunity for beetles to sense them [5,7], we do not know if the beetles use or act on this information. For instance, we would like to know if specific hydrocarbons elicit specific behaviors. Additionally, we would like to know if we can we change the outcome of agonistic or courtship interactions by changing the hydrocarbon mixture. Finally, we think that CHCs are only part of the story. Beetles are likely communicating in other ways, including visual, sound, vibration, and tactile signals [5,7–9,27,44]. Additional research is required to explore the interactions among these possible modes of communication.

Rhinoceros beetles are some of the largest insects and they wield one of the largest animal weapons relative to body size [4–7]. Our results suggest that their complex mating behavior might be partially explained by mixtures of chemical signals found on their surfaces. Future work will try to understand exactly how these signals are related to aggressive and mating behavior.

## Supporting information

S1 Fig Mass spectra examples. Example mass spectra for gas chromatogram Peaks 1–37 from *Trypoxylus dichotomus*. Spectra obtained from samples collected by washing elytra with hexanes.
(PDF)

S1 File Individual beetle data. Measurement and CHC proportion data from the individual beetles used in all of the analyses in this study.
(XLSX)
