# Supplementary material for: Rhinoceros beetle (Trypoxylus dichotomus) cuticular hydrocarbons contain information about body size and sex
Source: PLoS One. 2024 Mar 14;19(3):e0299796. doi: 10.1371/journal.pone.0299796 (PMC10939270; doi:10.1371/journal.pone.0299796)
Supplement: S1 Fig — Example mass spectra for gas chromatogram Peaks 1–37 from Trypoxylus dichotomus. Spectra obtained from samples collected by washing elytra with hexanes. (PDF) [file pone.0299796.s001.pdf]

Supplementary Figure 1 - Example mass spectra for gas chromatogram Peaks 1-37 from *Trypoxylus dichotomus*.

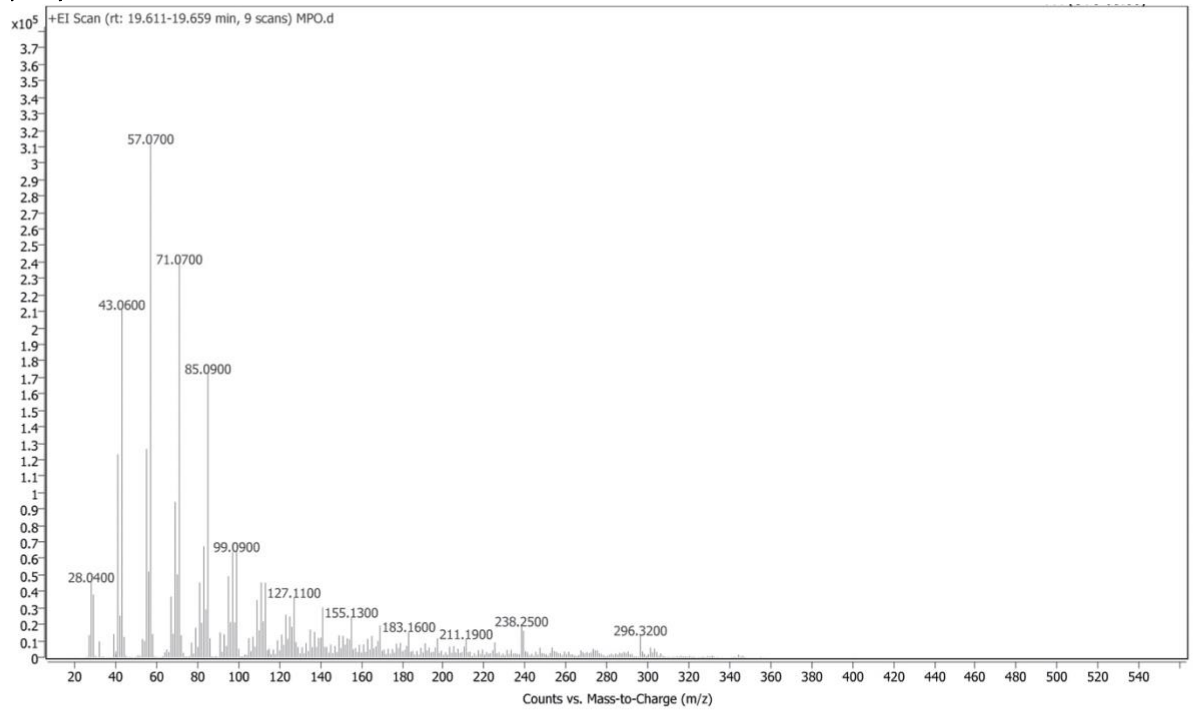

Peak 1

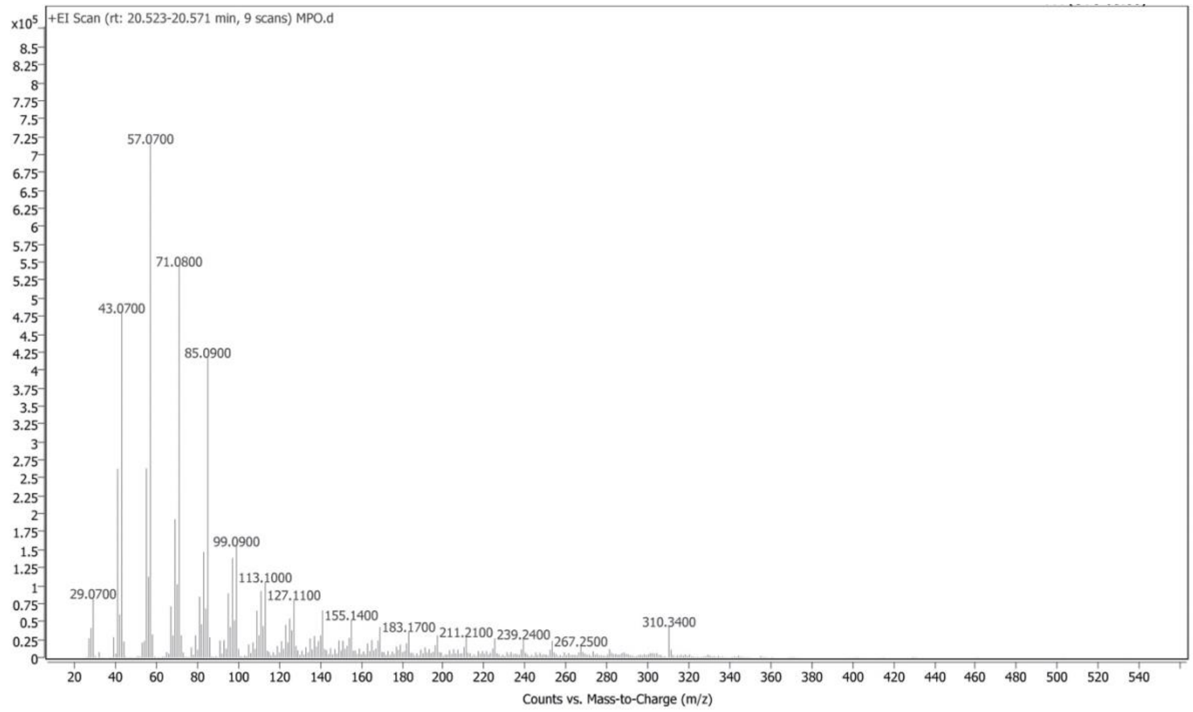

Peak 2

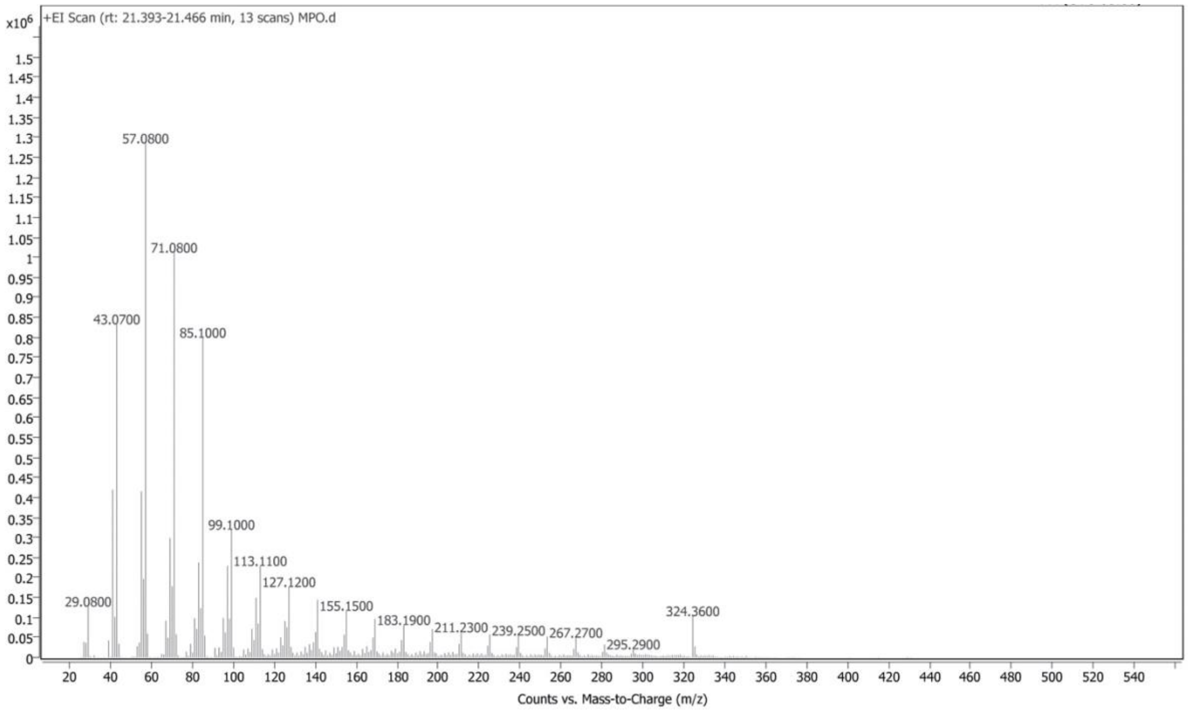

x10^6
+EI Scan (rt: 21.393-21.466 min, 13 scans) MPO.d
57.0800
71.0800
43.0700
85.1000
99.1000
113.1100
127.1200
155.1500
183.1900
211.2300
239.2500
267.2700
295.2900
324.3600
29.0800
Counts vs. Mass-to-Charge (m/z)

Peak 3

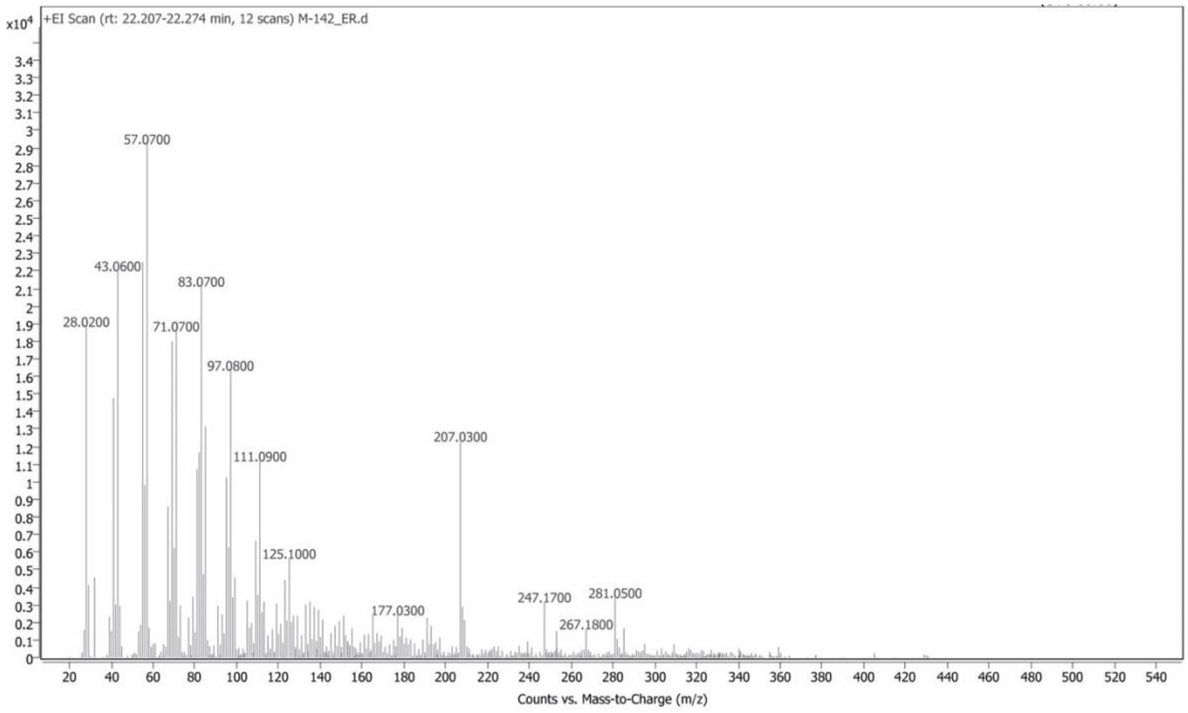

x10^4
+EI Scan (rt: 22.207-22.274 min, 12 scans) M-142_ER.d
57.0700
43.0600
83.0700
28.0200
71.0700
97.0800
207.0300
111.0900
125.1000
177.0300
247.1700
281.0500
267.1800
Counts vs. Mass-to-Charge (m/z)

Peak 4

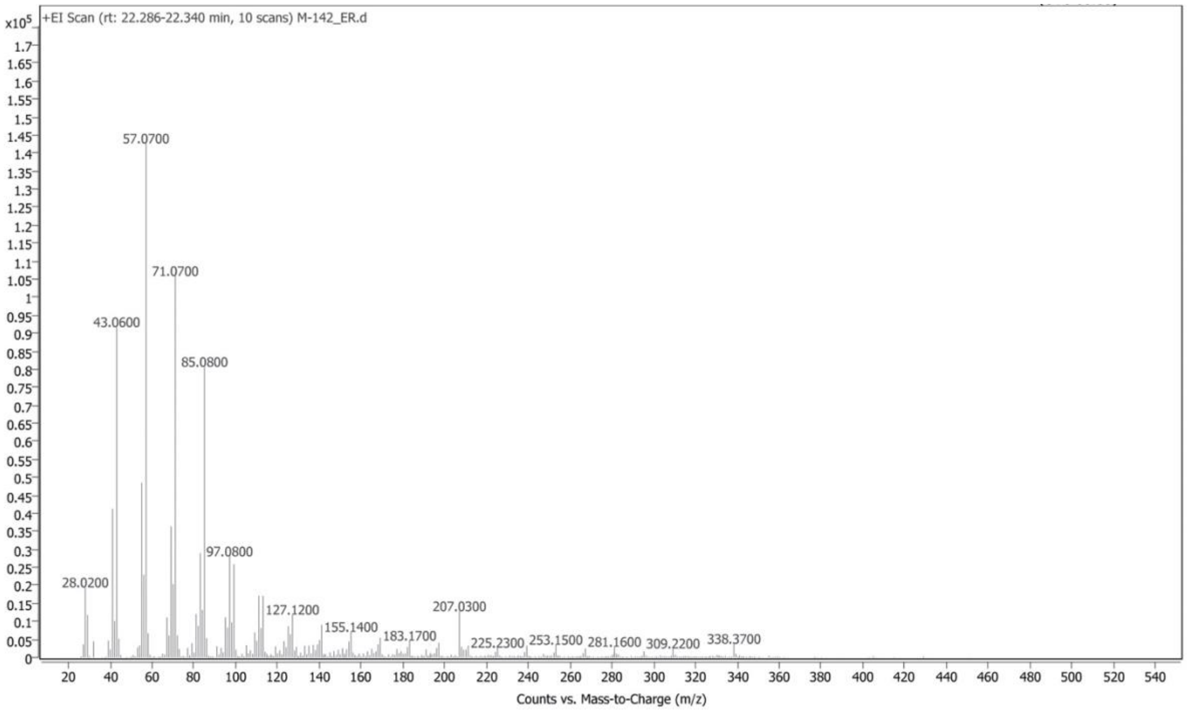
+EI Scan (rt: 22.286-22.340 min, 10 scans) M-142_ER.d
x10^5
57.0700
71.0700
43.0600
85.0800
97.0800
28.0200
127.1200
155.1400
183.1700
207.0300
225.2300
253.1500
281.1600
309.2200
338.3700
Counts vs. Mass-to-Charge (m/z)

Peak 5

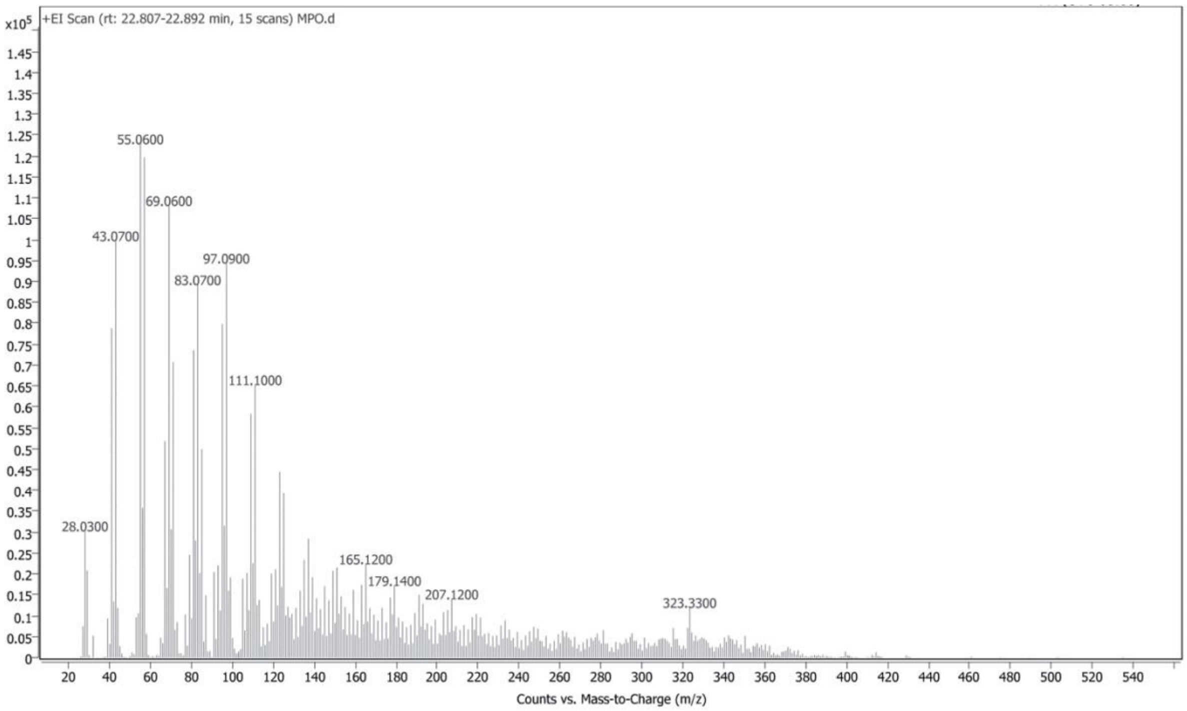
+EI Scan (rt: 22.807-22.892 min, 15 scans) MPO.d
x10^5
55.0600
69.0600
43.0700
97.0900
83.0700
111.1000
28.0300
165.1200
179.1400
207.1200
323.3300
Counts vs. Mass-to-Charge (m/z)

Peak 6

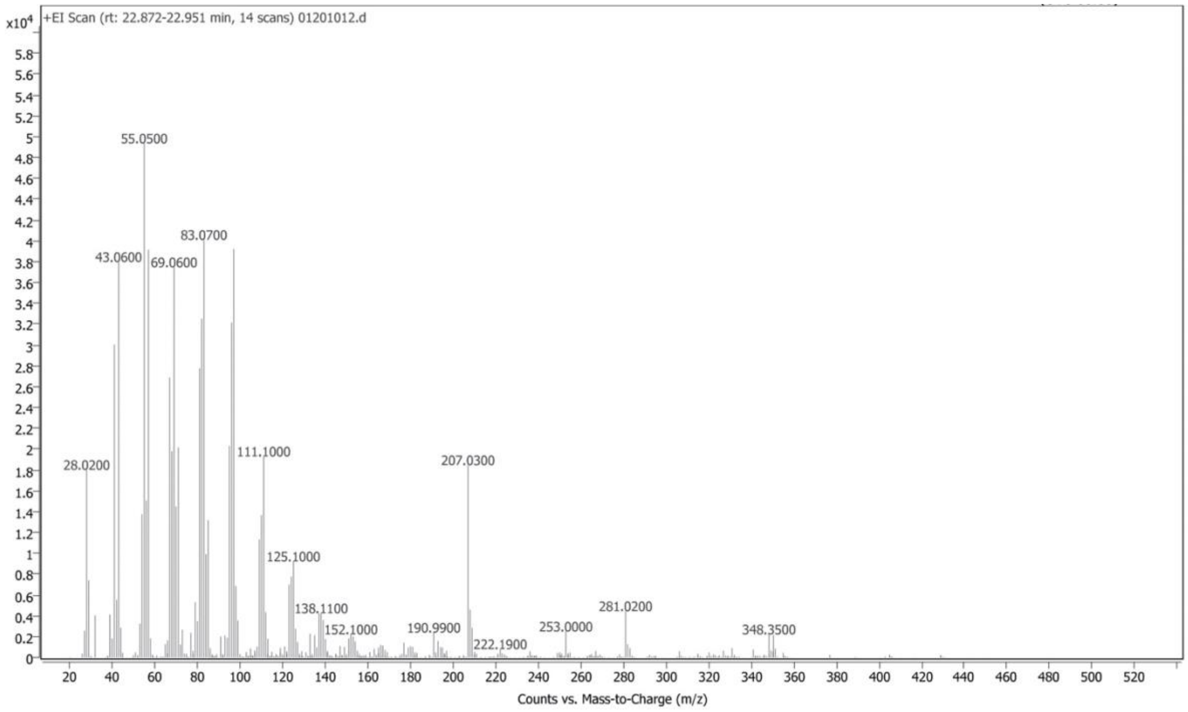
+EI Scan (rt: 22.872-22.951 min, 14 scans) 01201012.d
x10^4
55.0500
83.0700
43.0600
69.0600
111.1000
28.0200
207.0300
125.1000
138.1100
152.1000
190.9900
222.1900
253.0000
281.0200
348.3500
Counts vs. Mass-to-Charge (m/z)

Peak 7

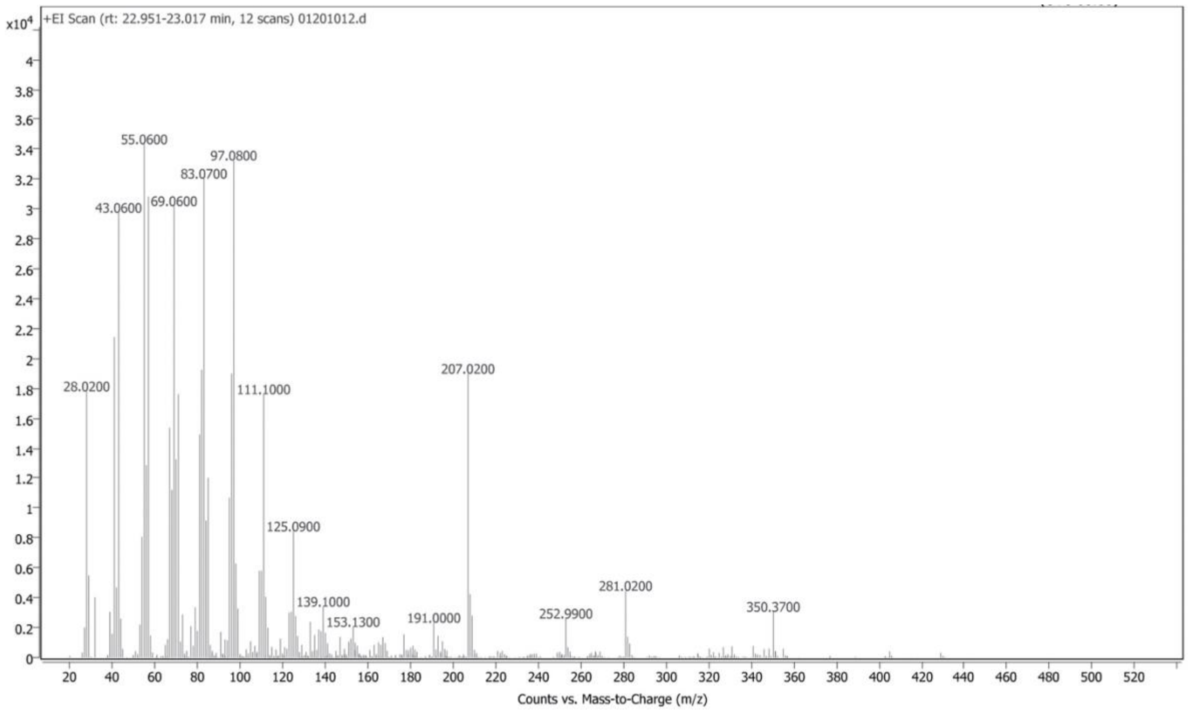
+EI Scan (rt: 22.951-23.017 min, 12 scans) 01201012.d
x10^4
55.0600
97.0800
83.0700
43.0600
69.0600
28.0200
111.1000
207.0200
125.0900
139.1000
153.1300
191.0000
252.9900
281.0200
350.3700
Counts vs. Mass-to-Charge (m/z)

Peak 8

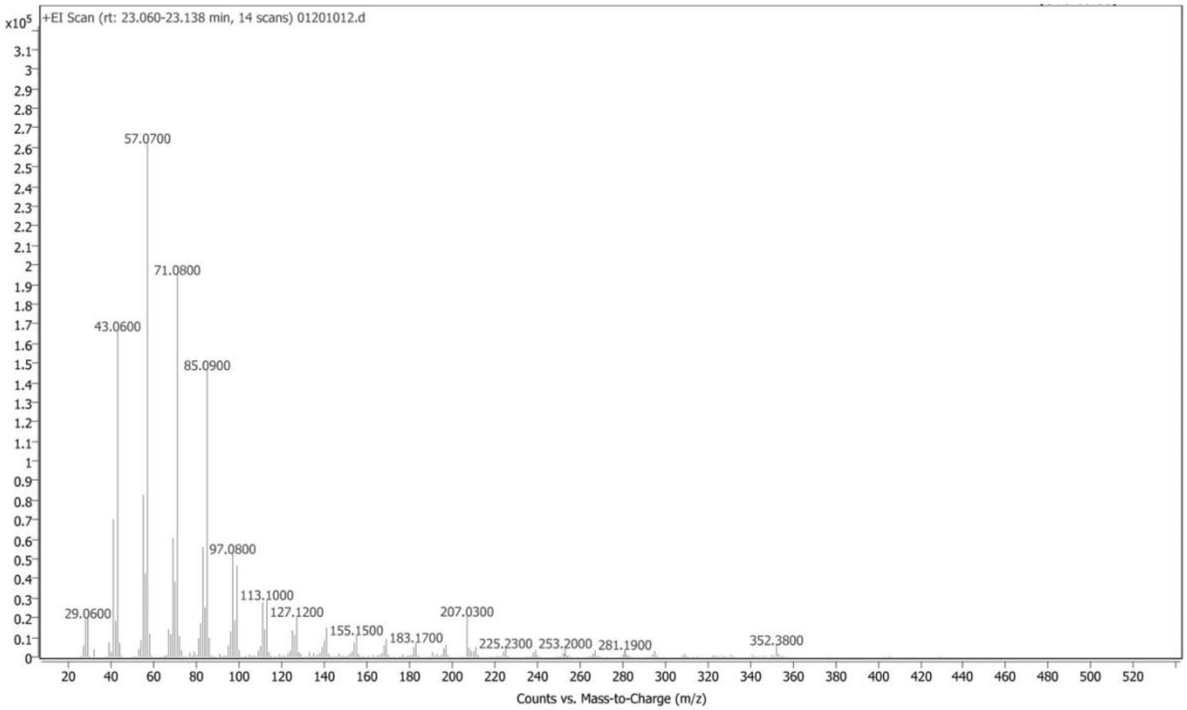

Peak 9

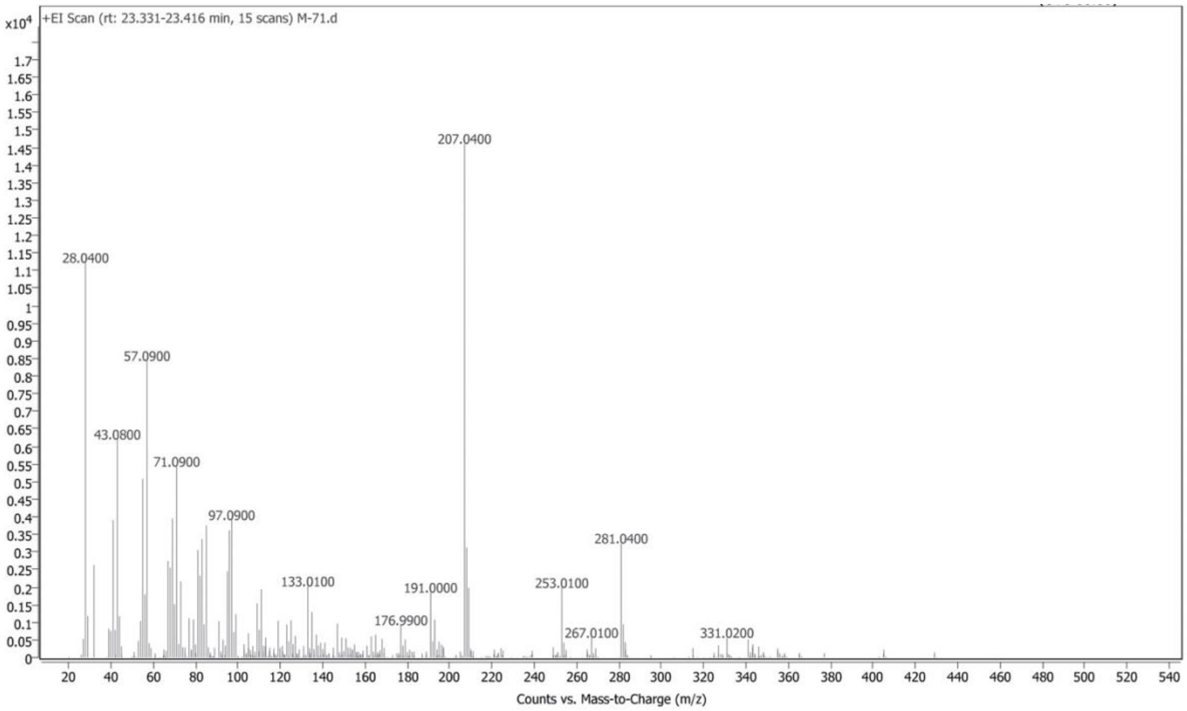

Peak 10

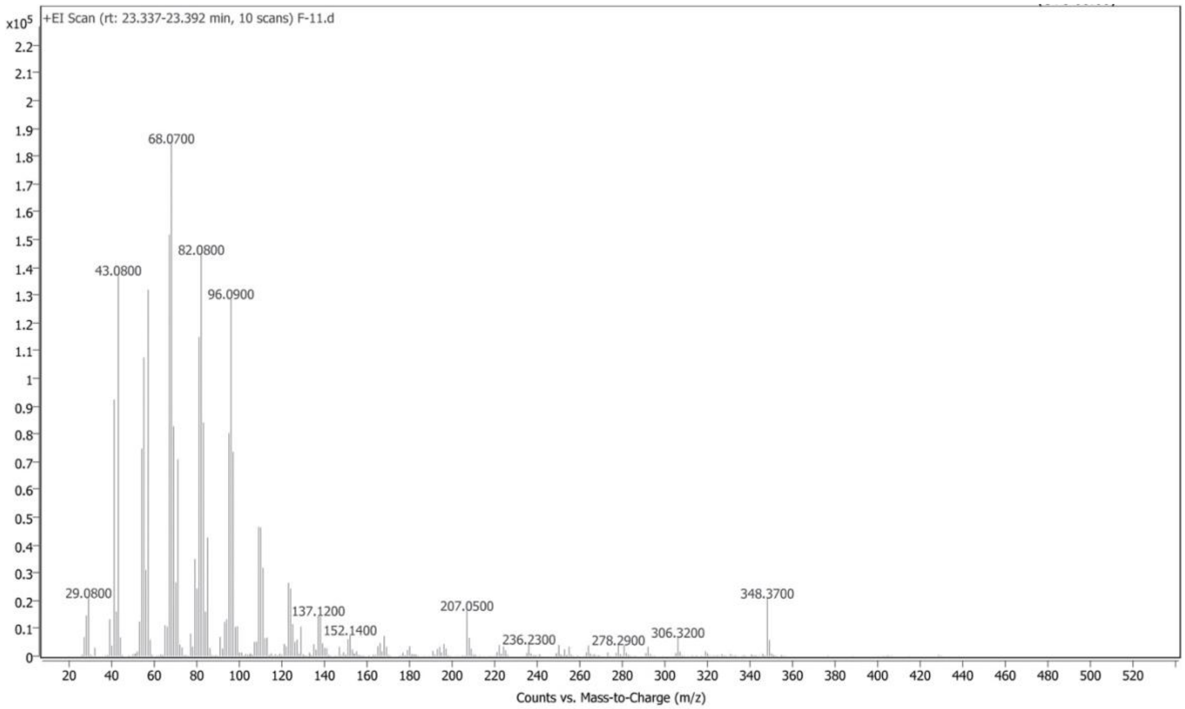

Peak 11

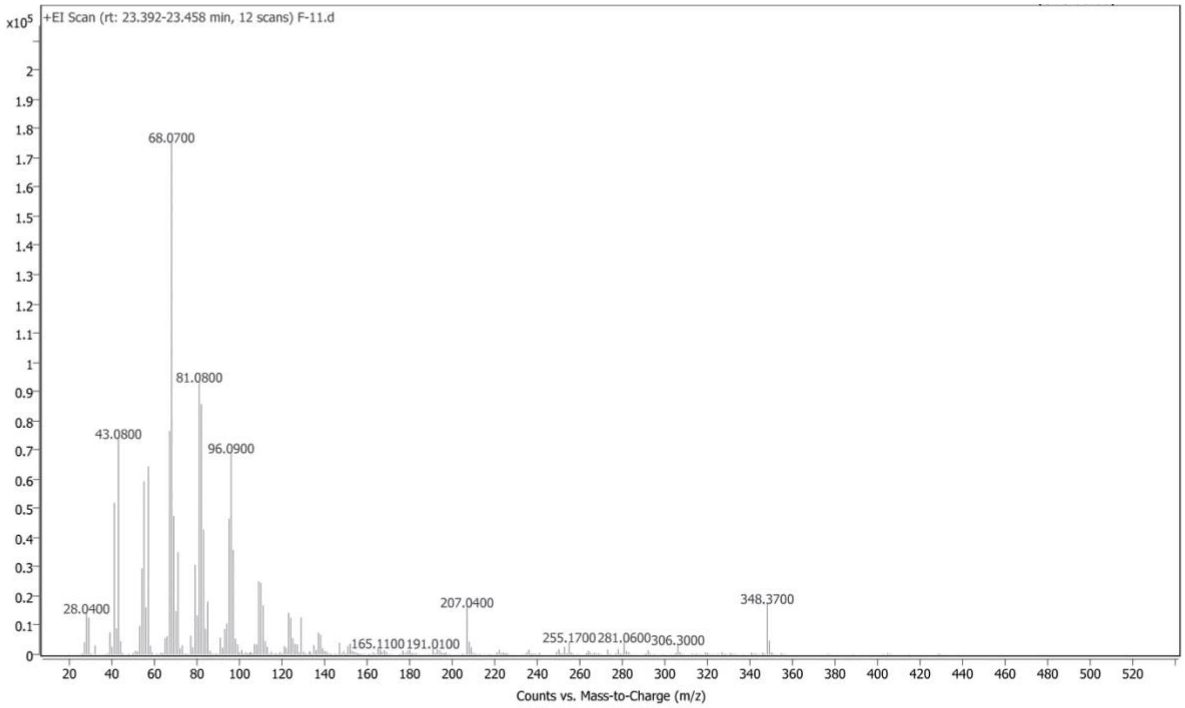

Peak 12

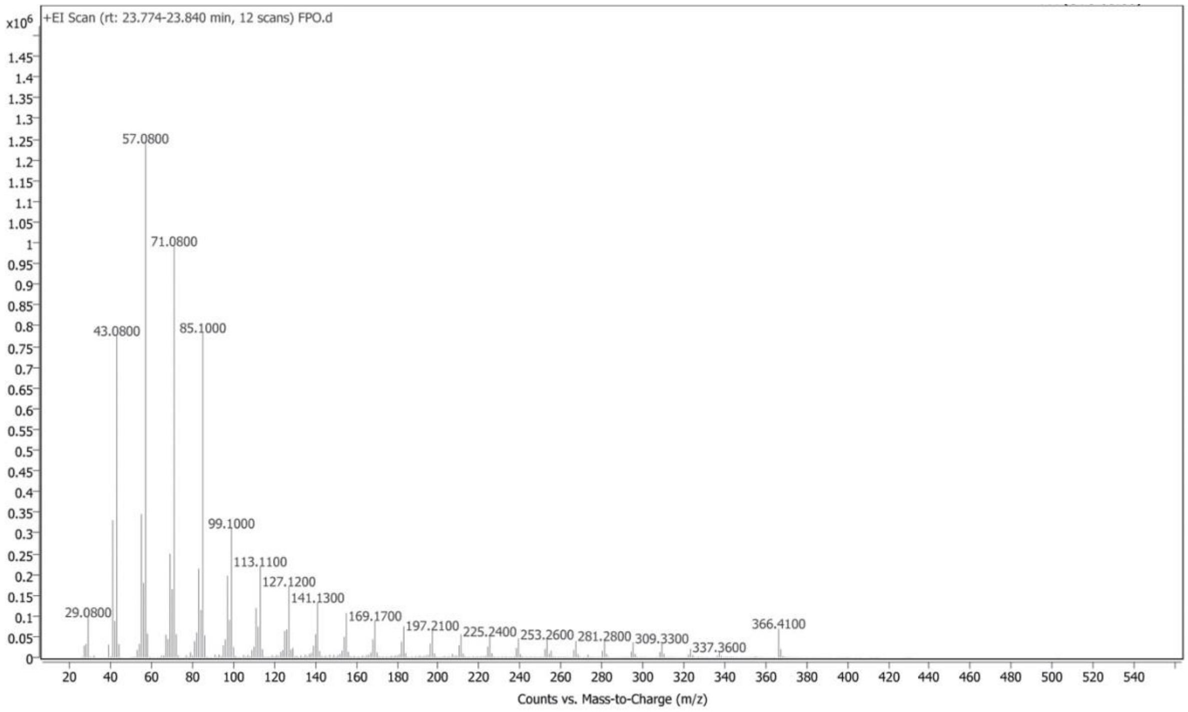

Peak 13

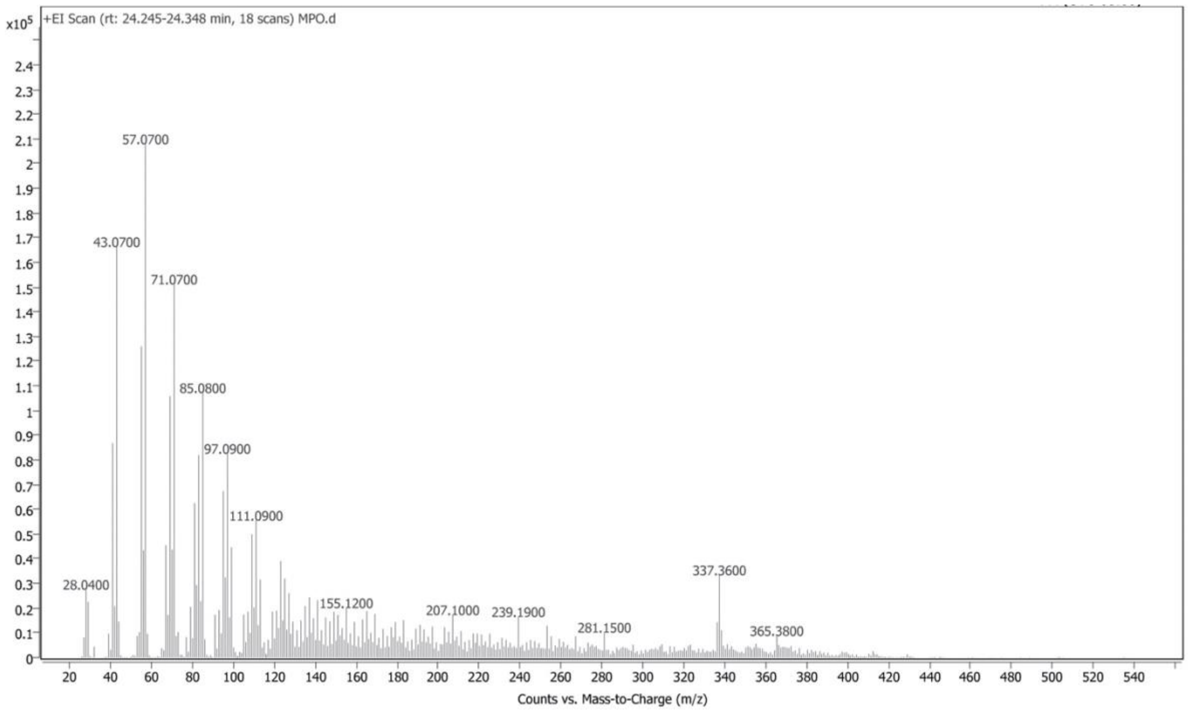

Peak 14

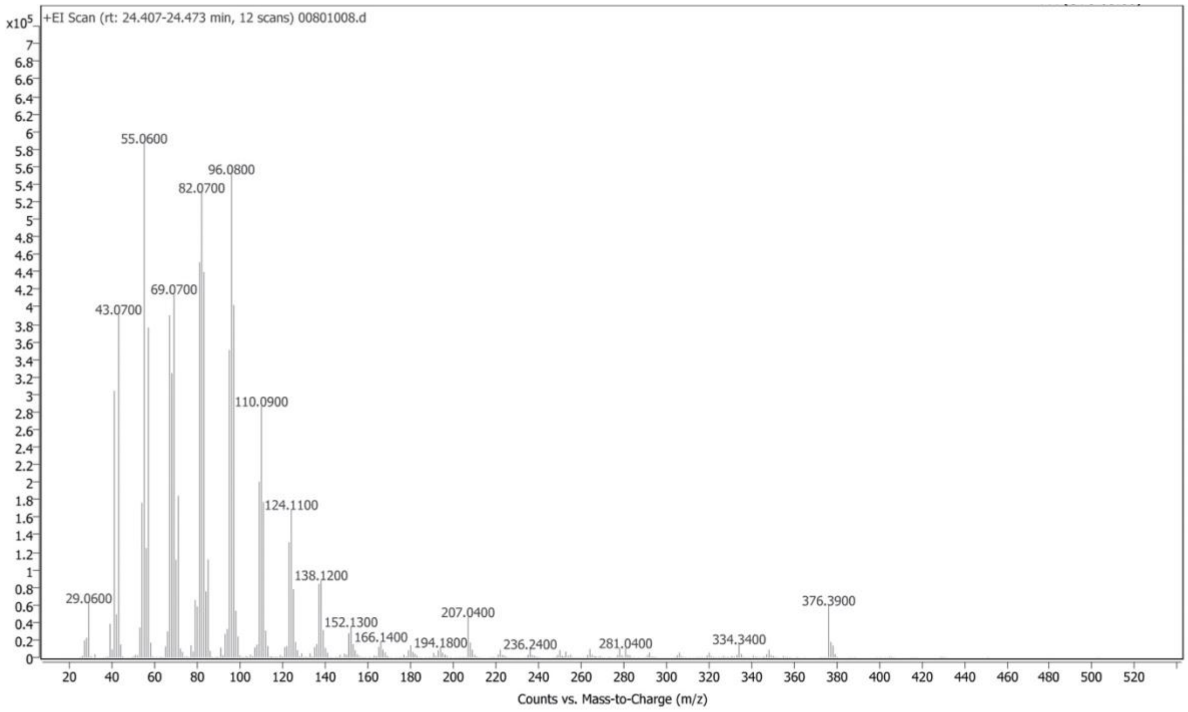

Peak 15

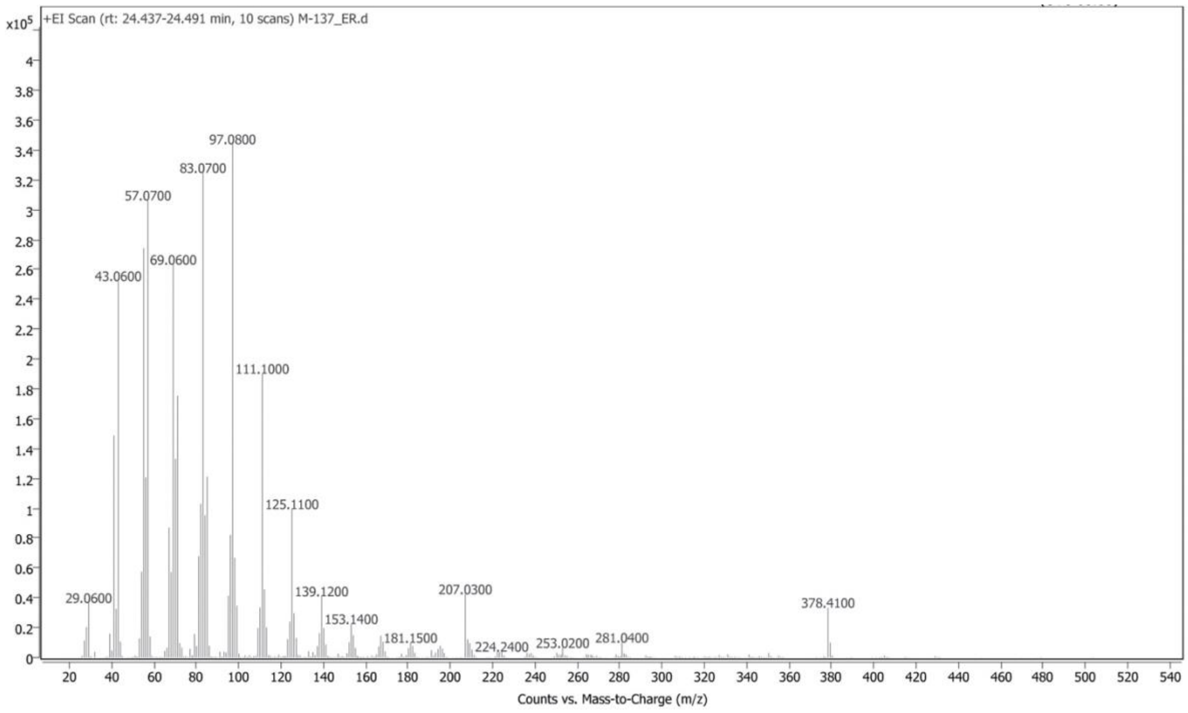

Peak 16

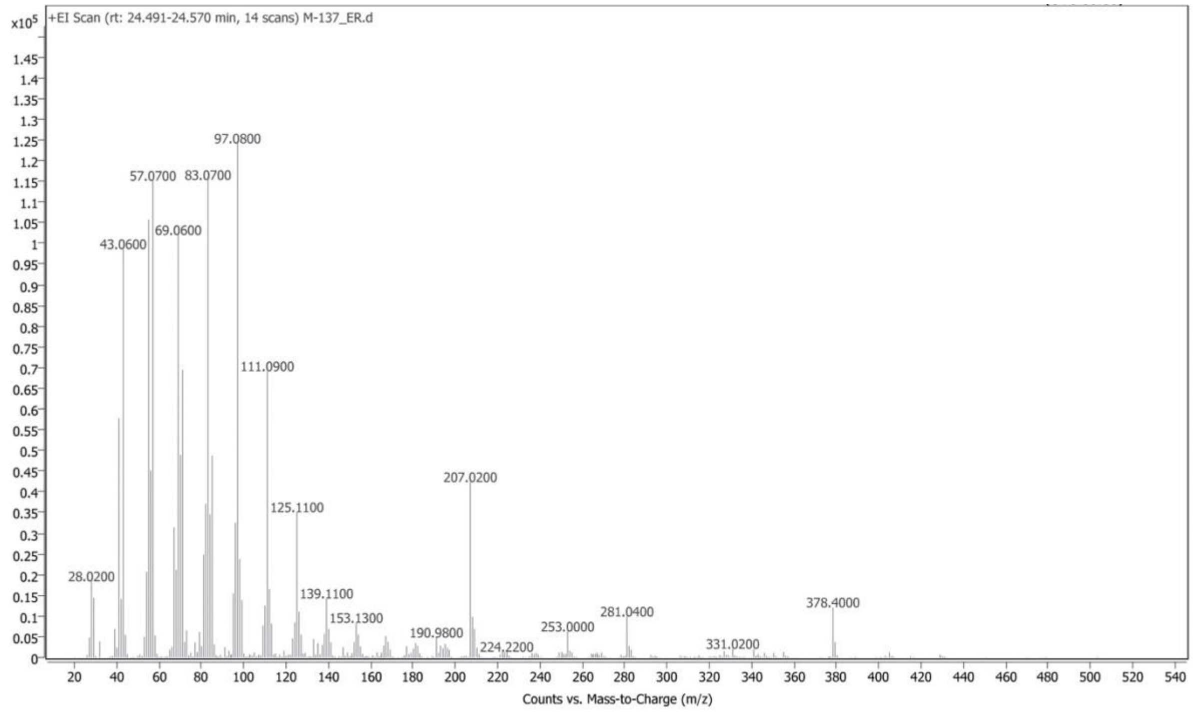

Peak 17

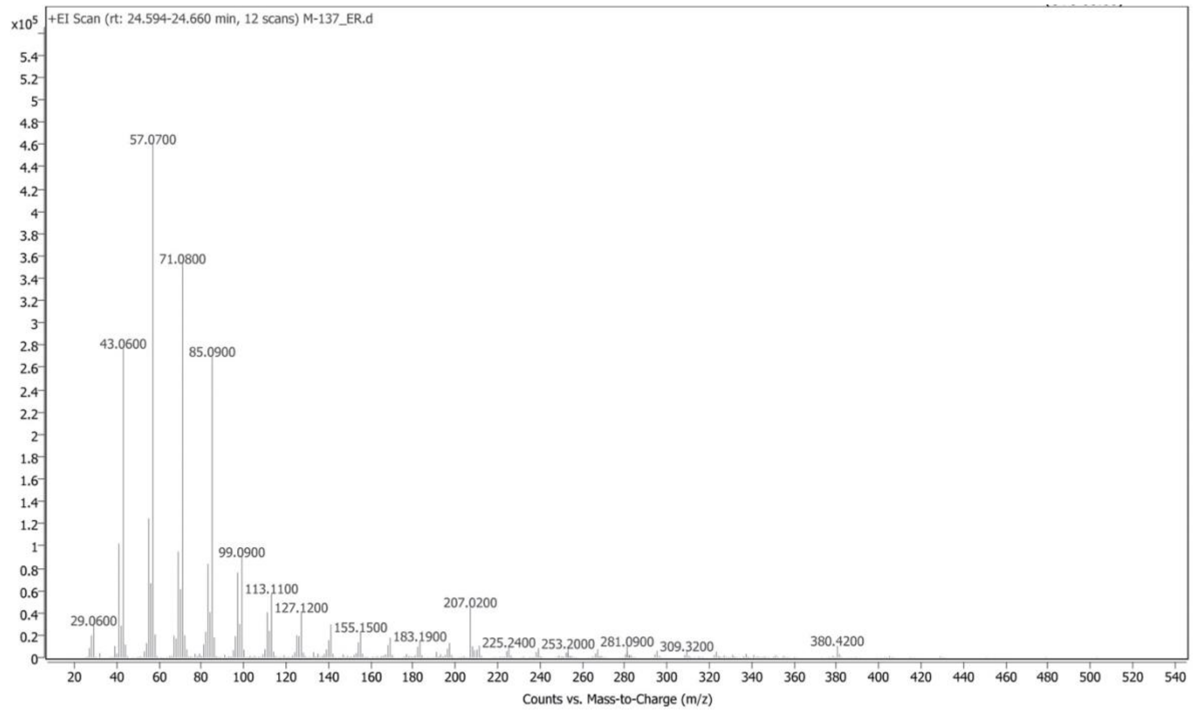

Peak 18

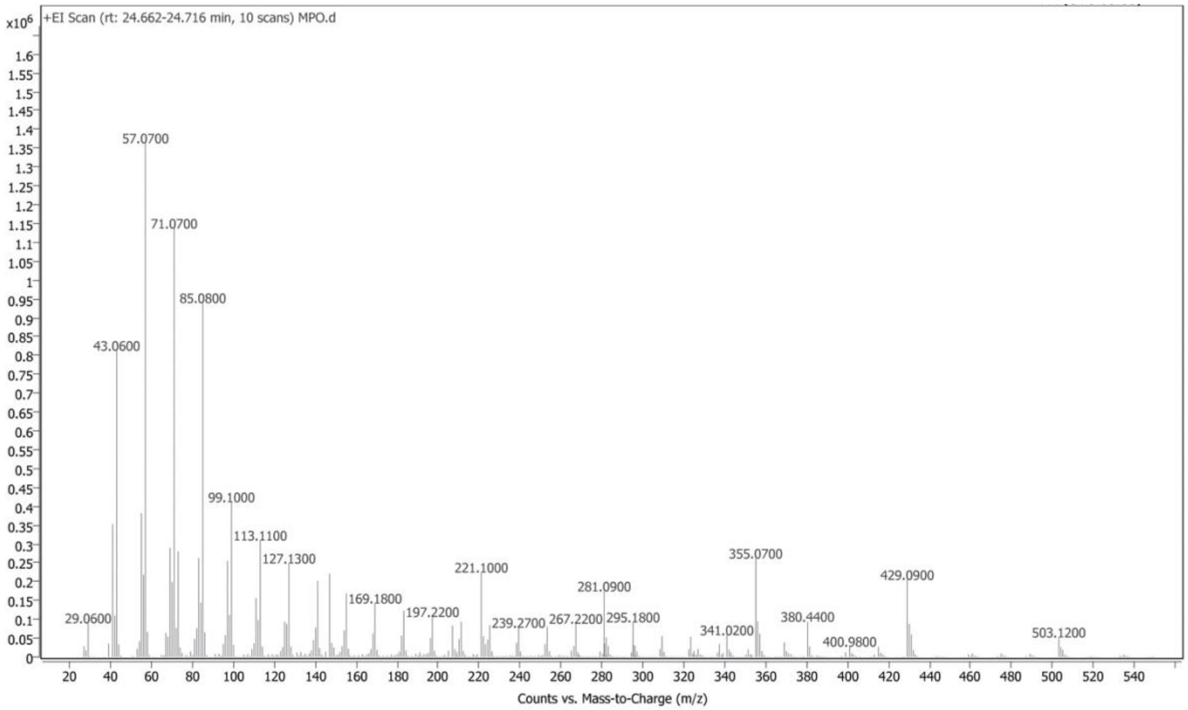

Peak 19

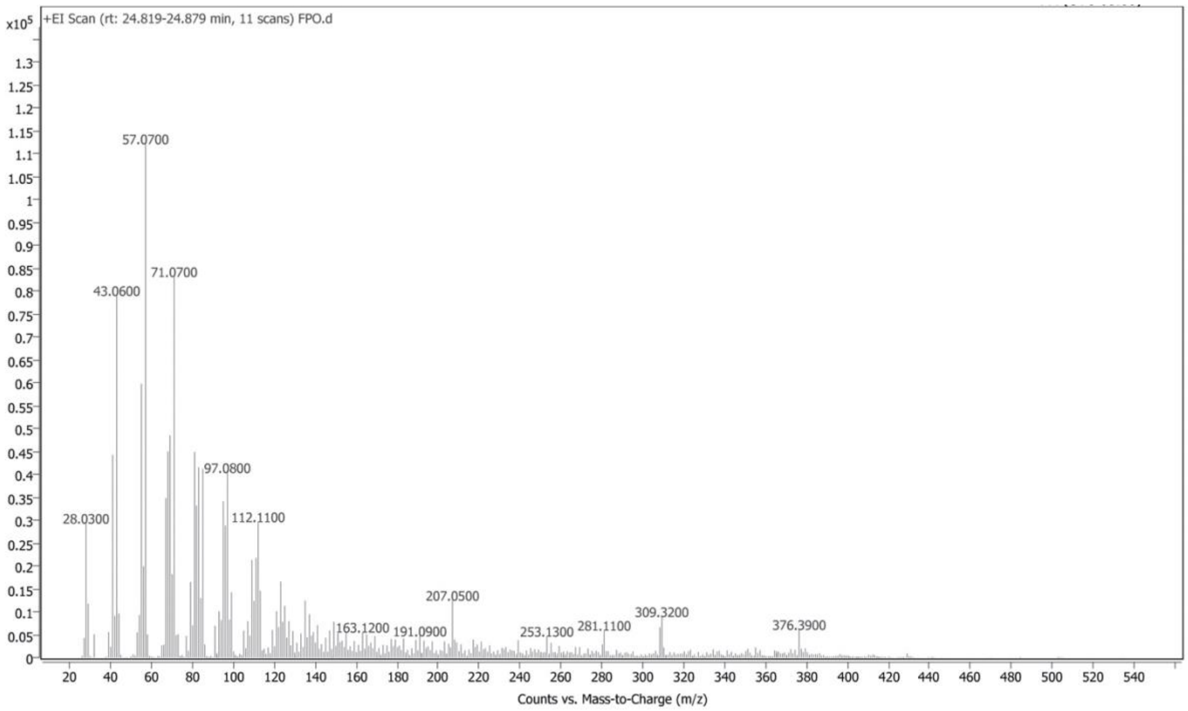

Peak 20

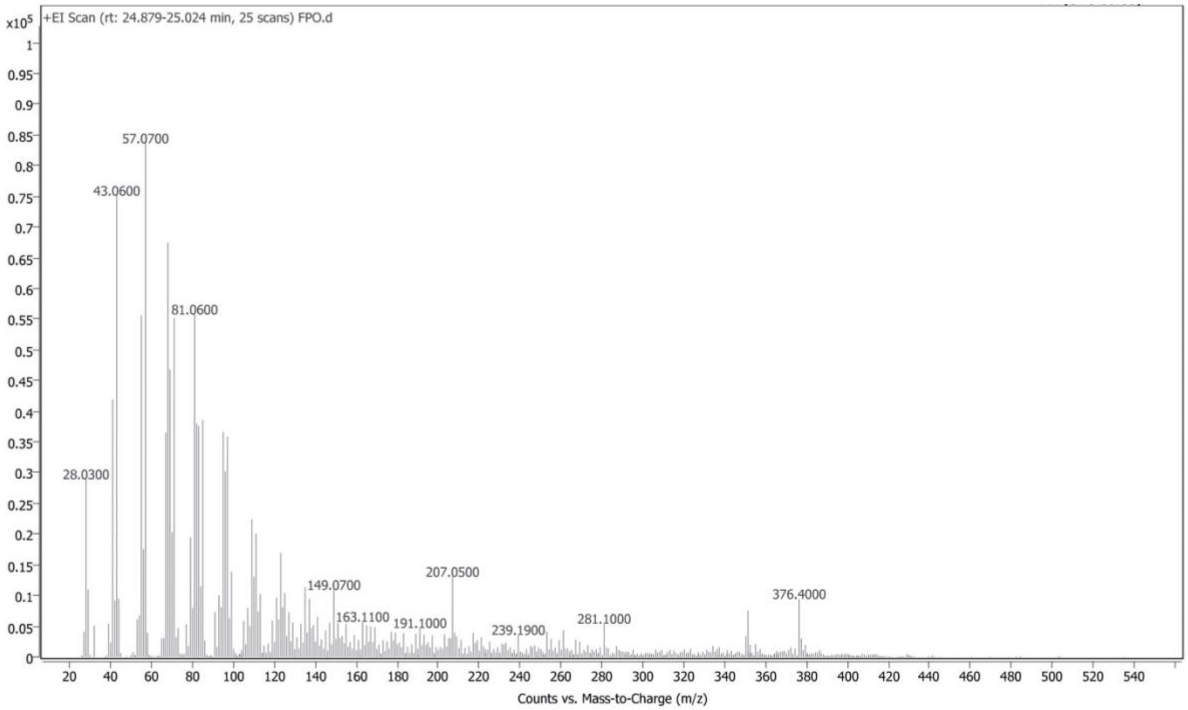

Peak 21

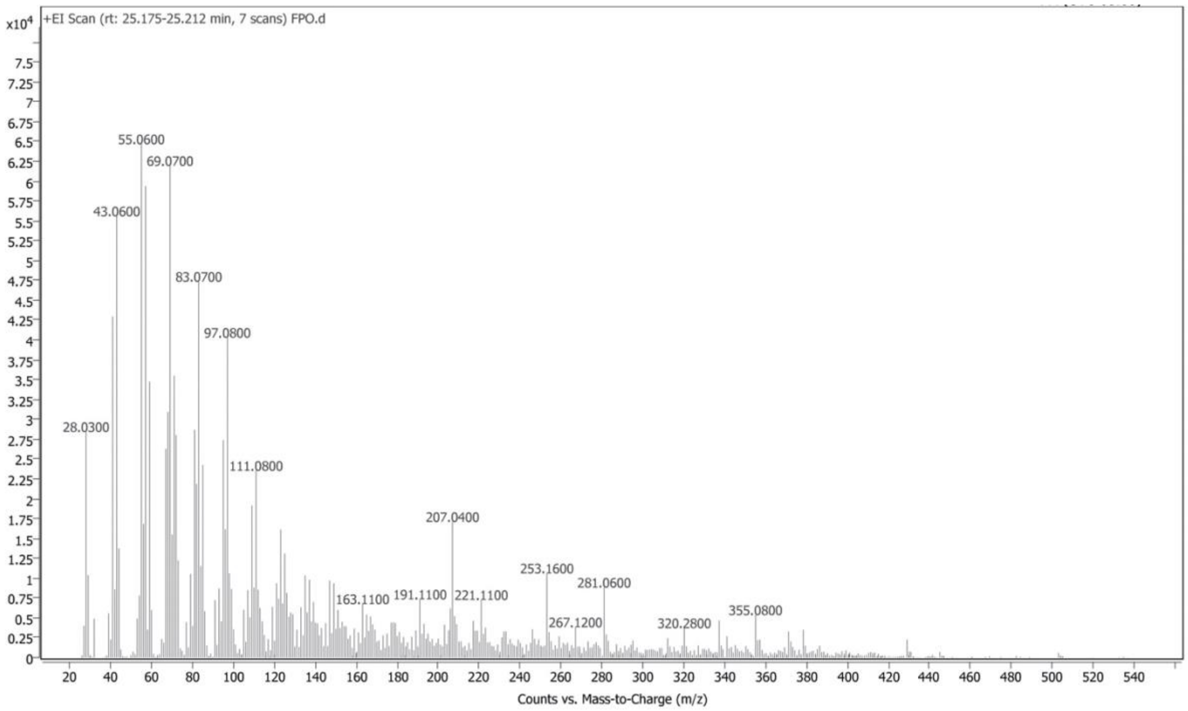

Peak 22

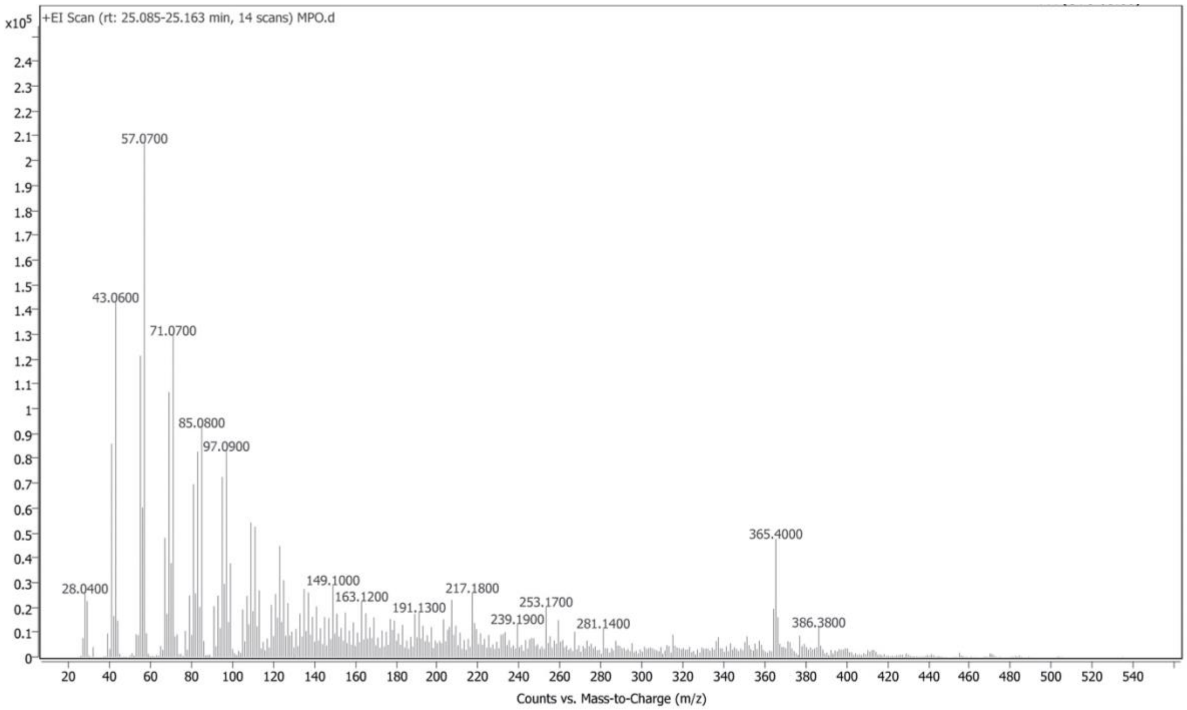

Peak 23

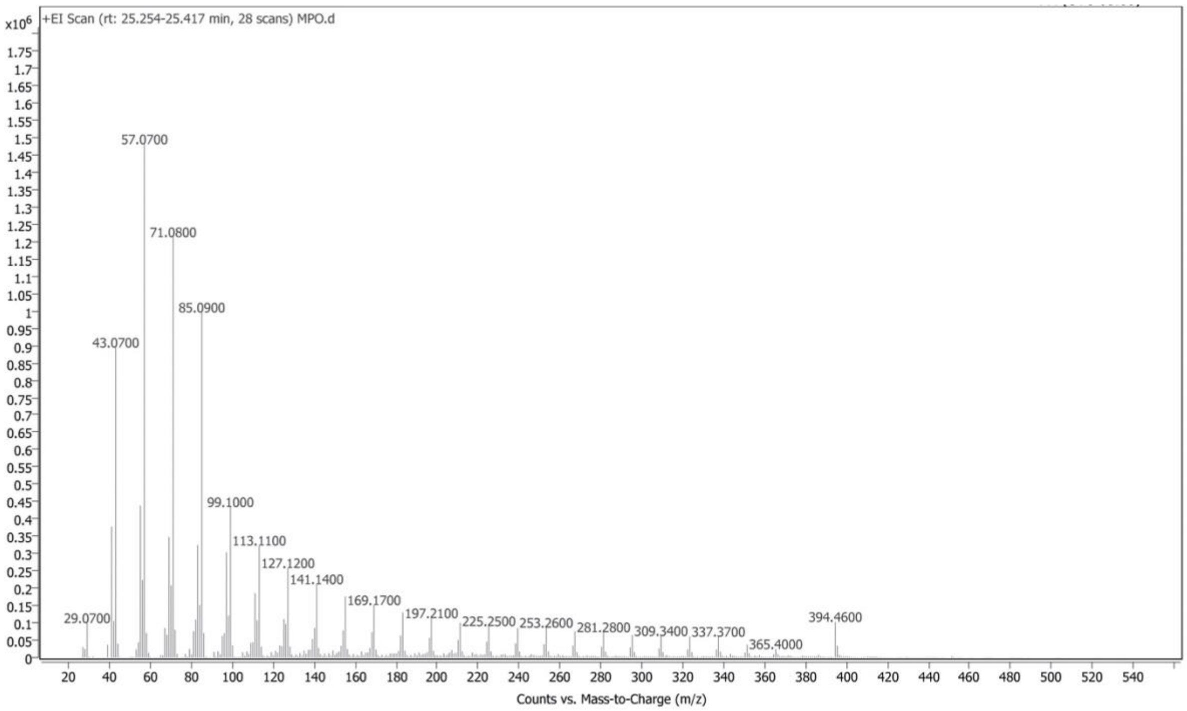

Peak 24

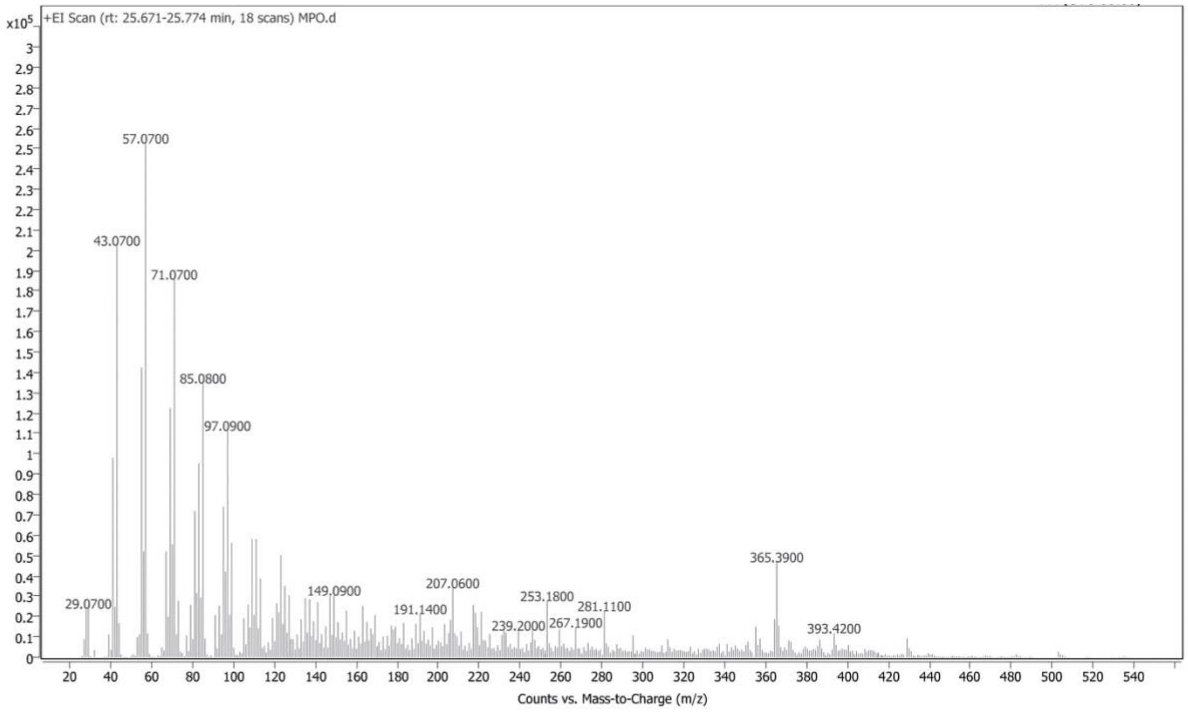

Peak 25

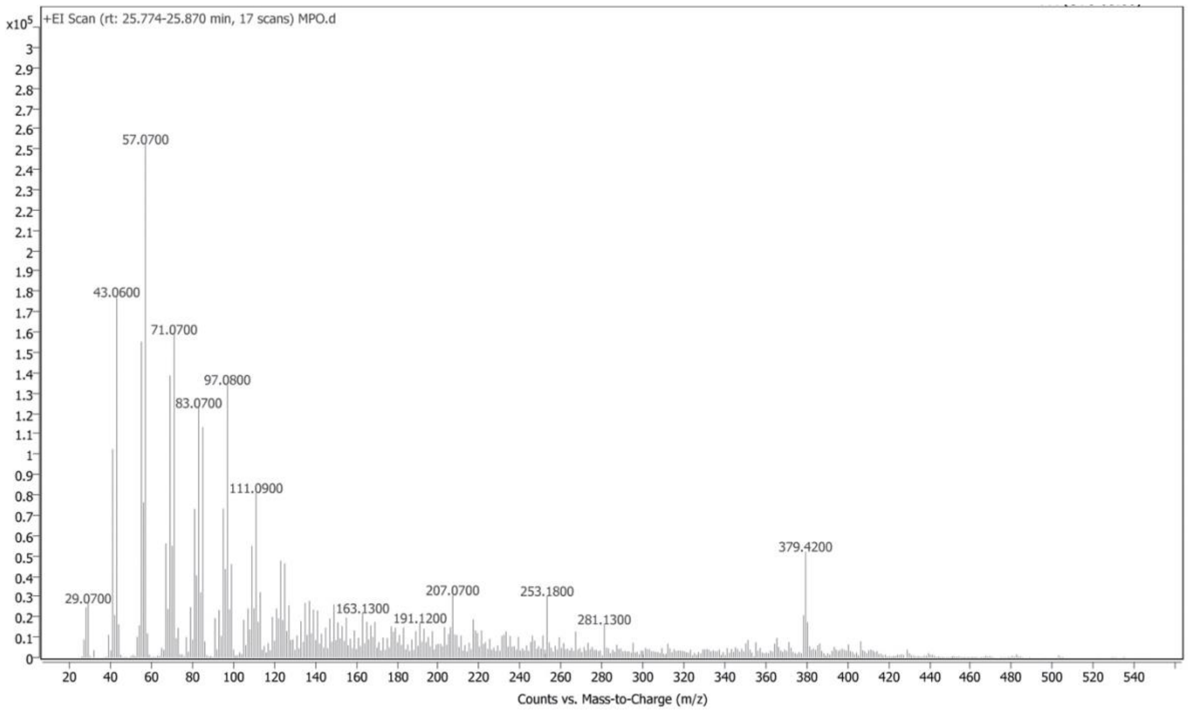

Peak 26

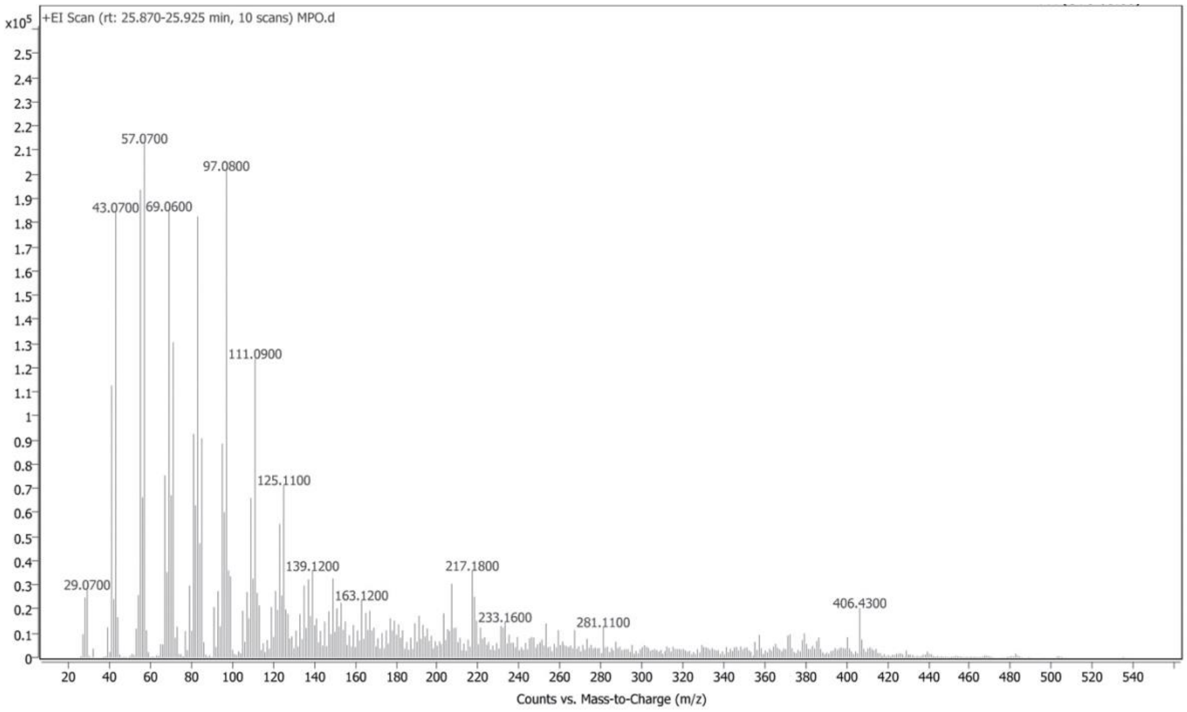

Peak 27

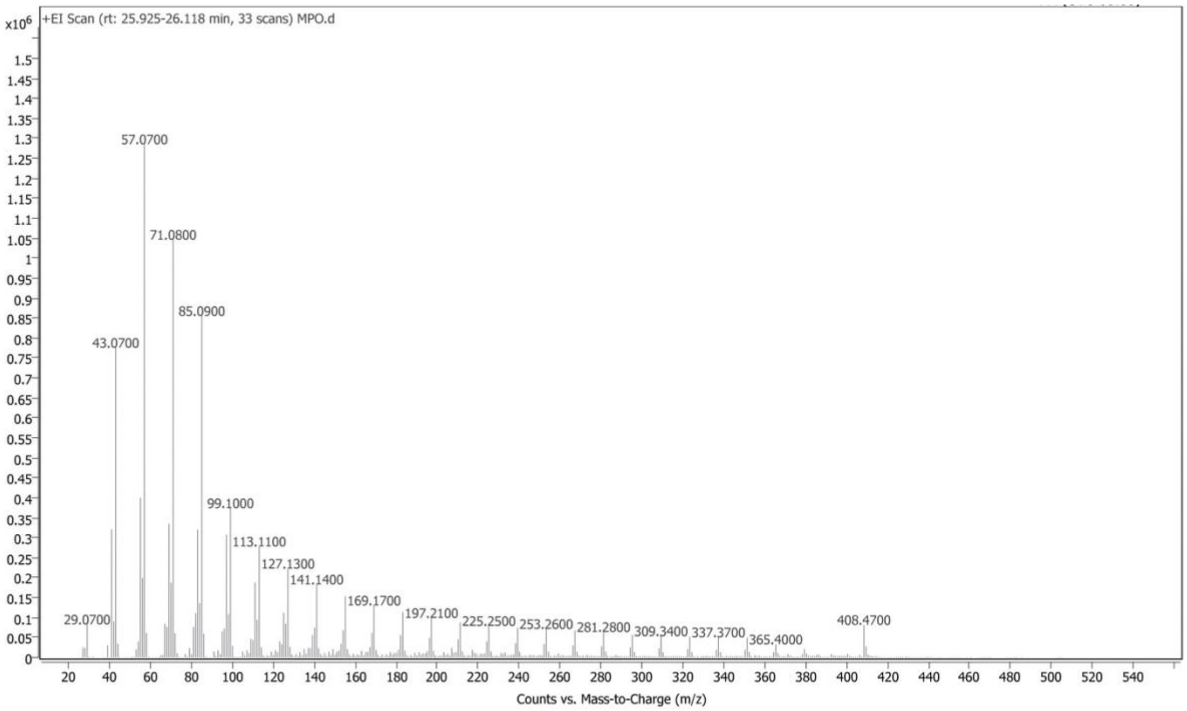

Peak 28

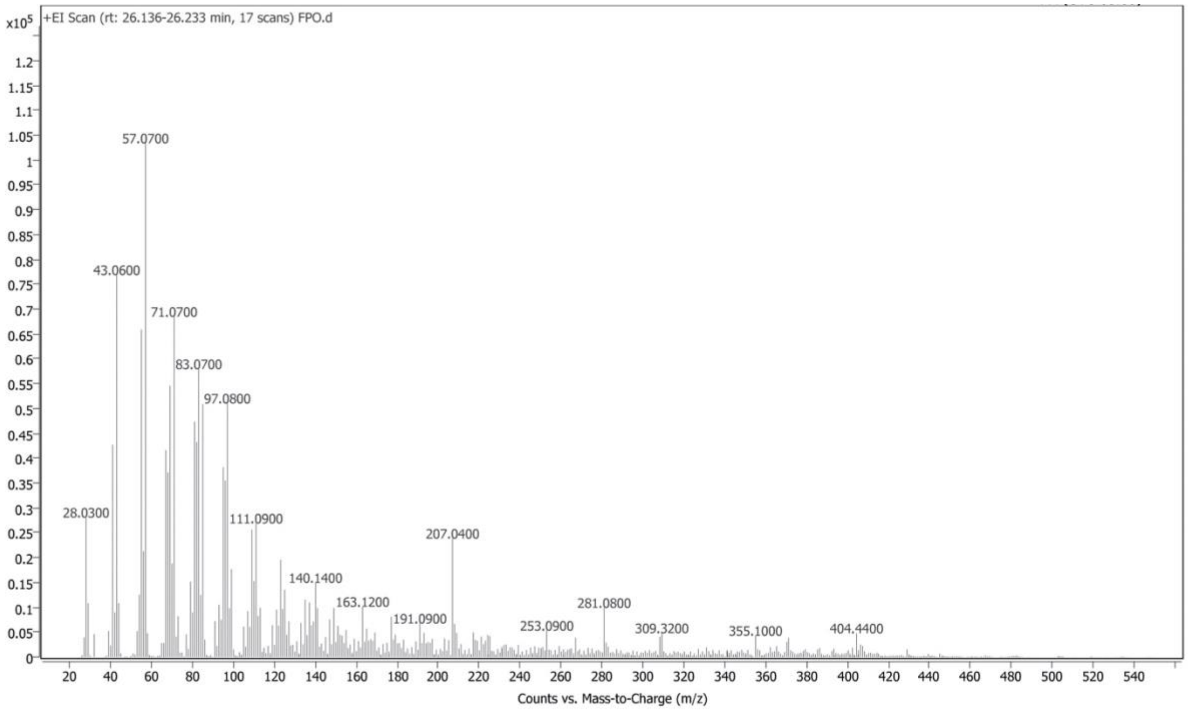

Peak 29

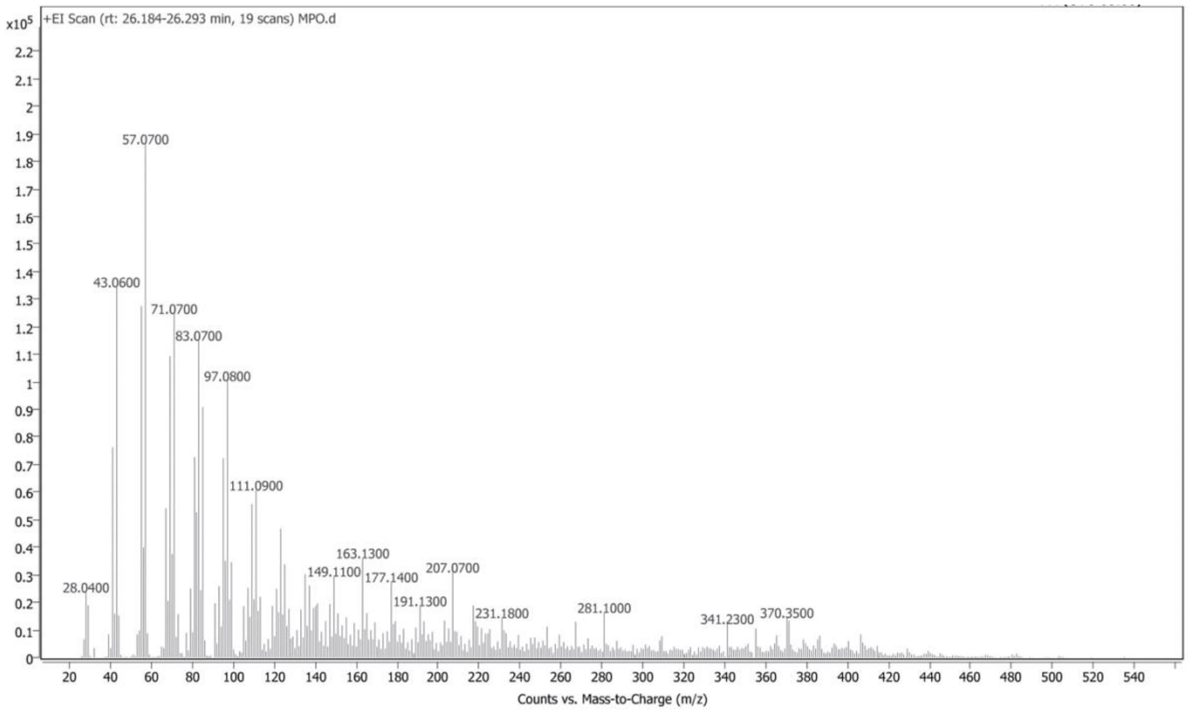

Peak 30

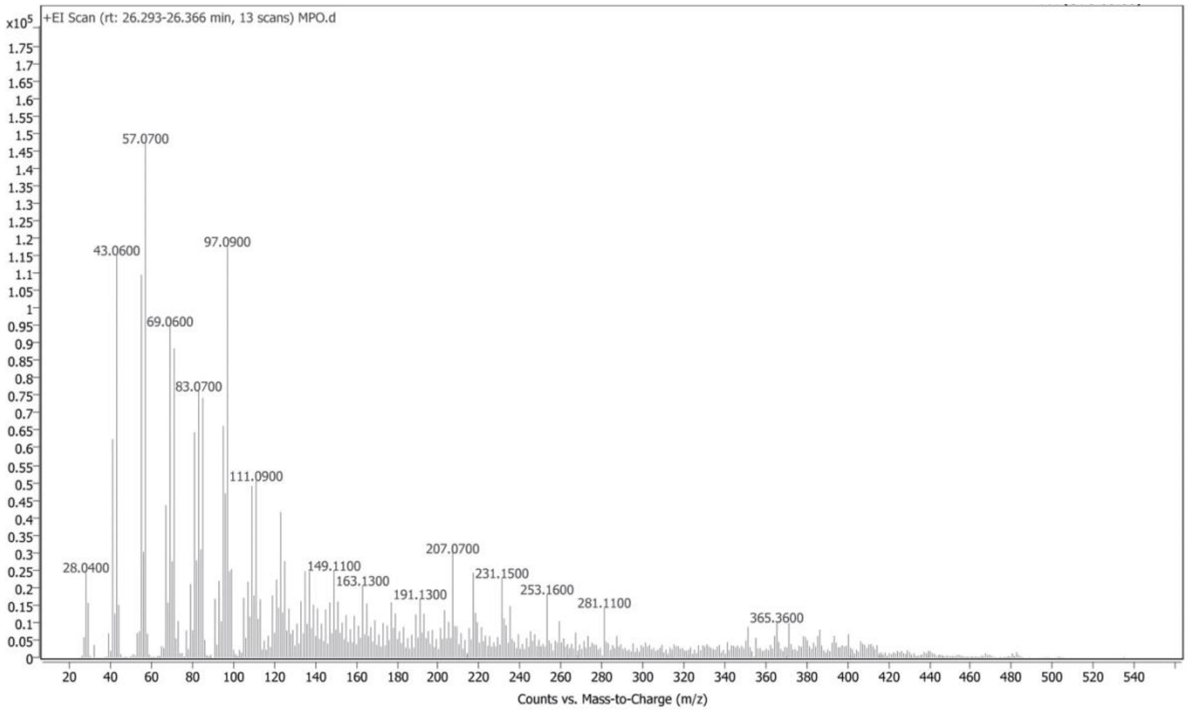

Peak 31

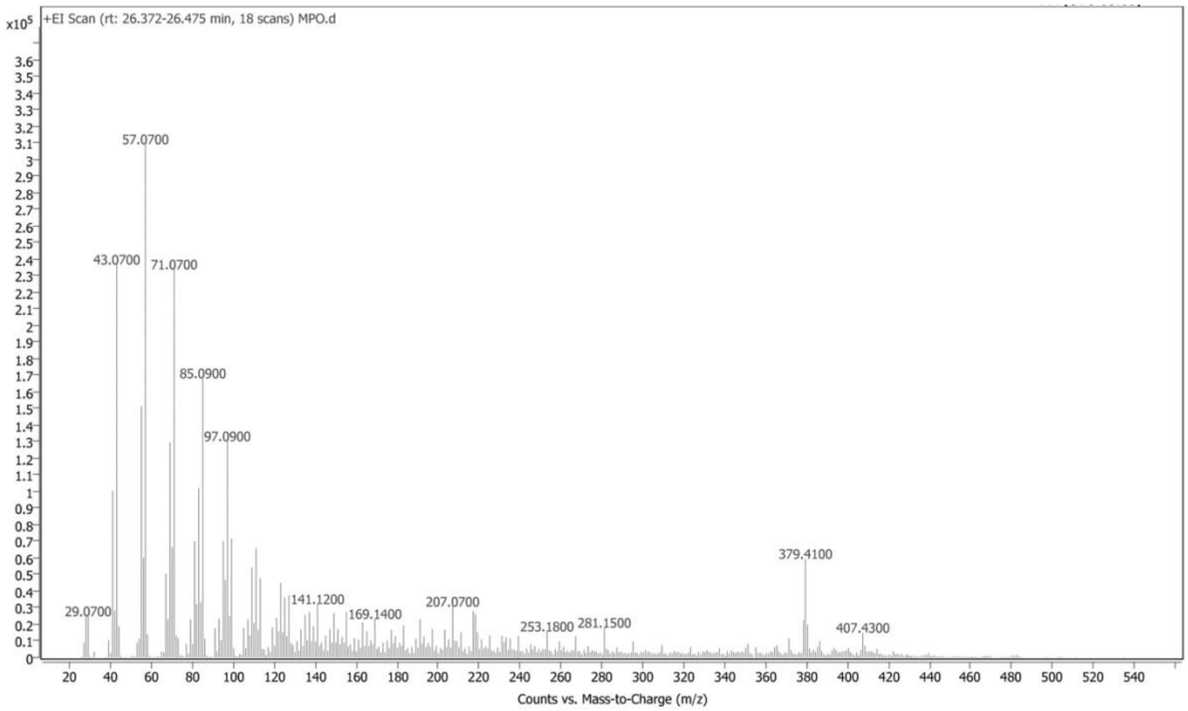

Peak 32

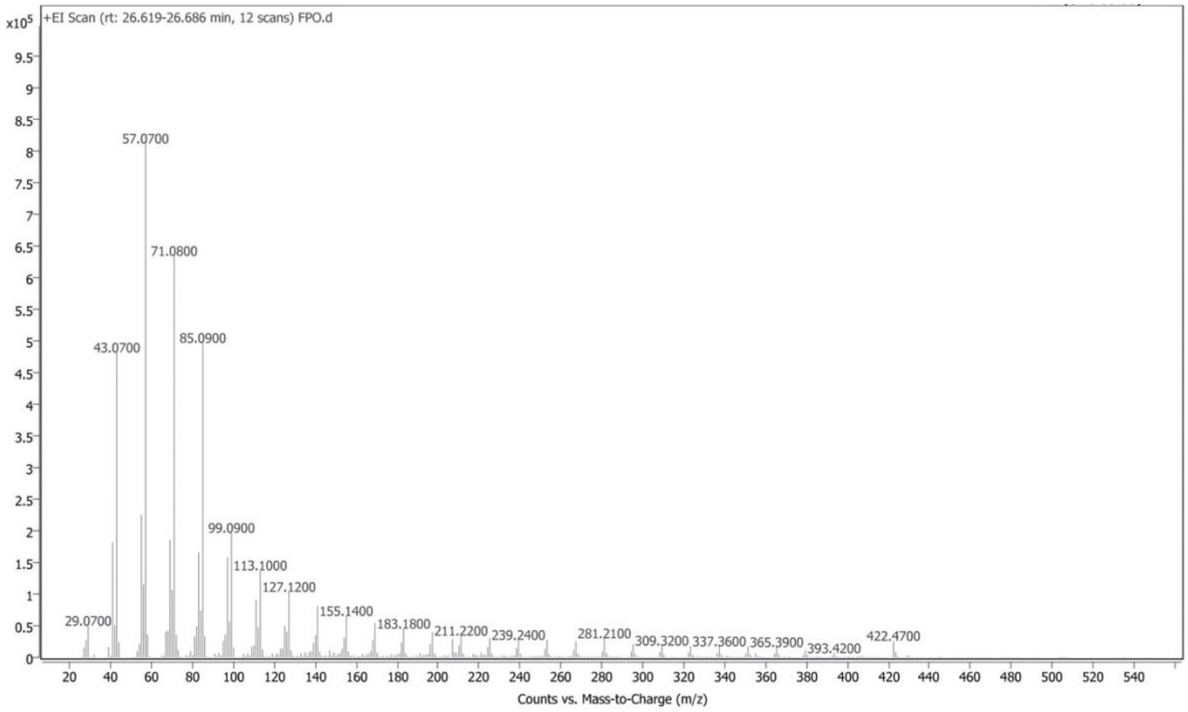

Peak 33

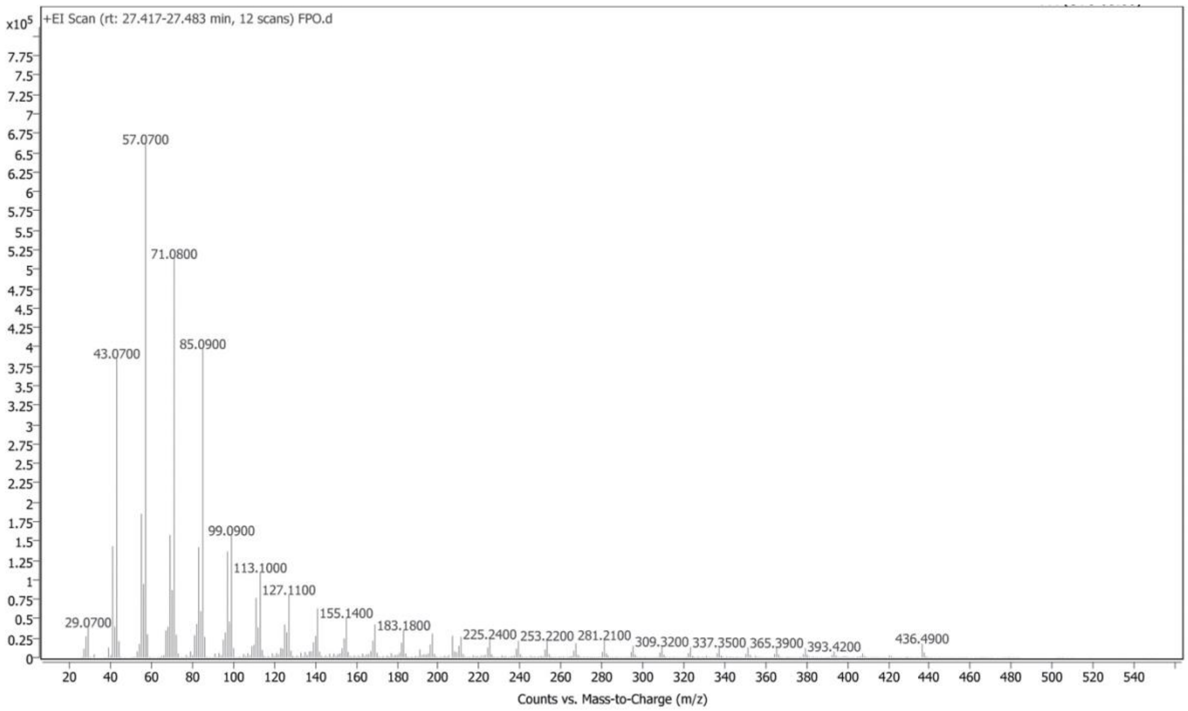

Peak 34

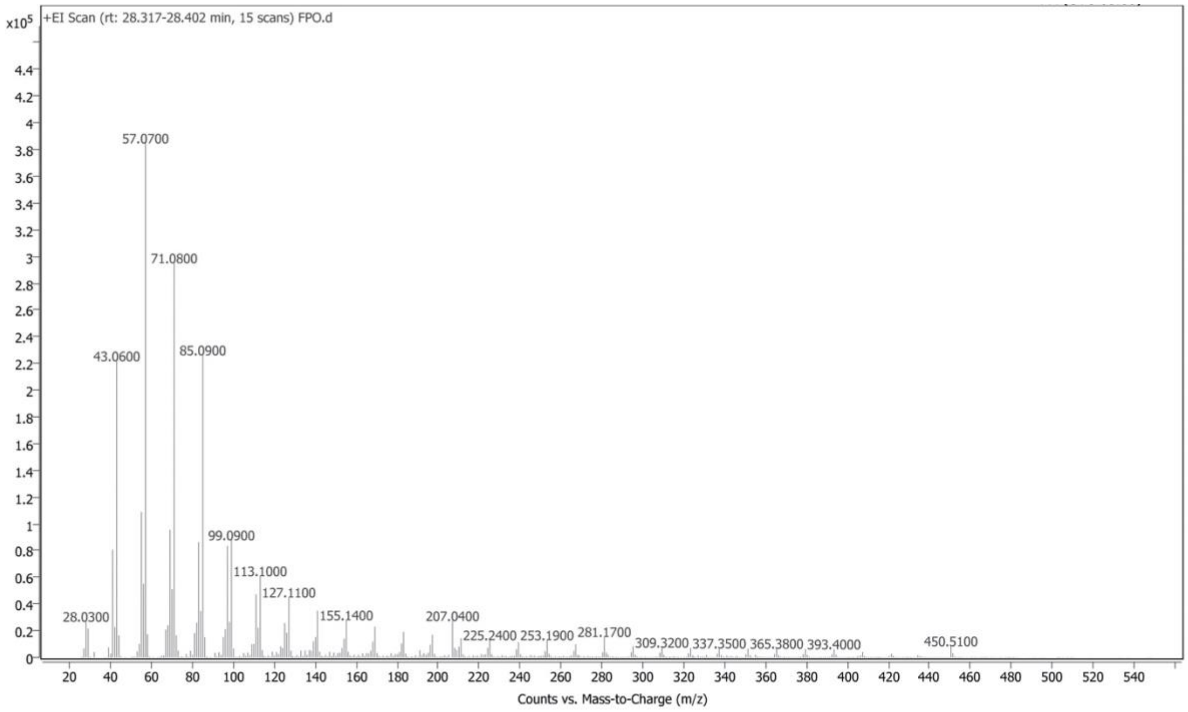

Peak 35

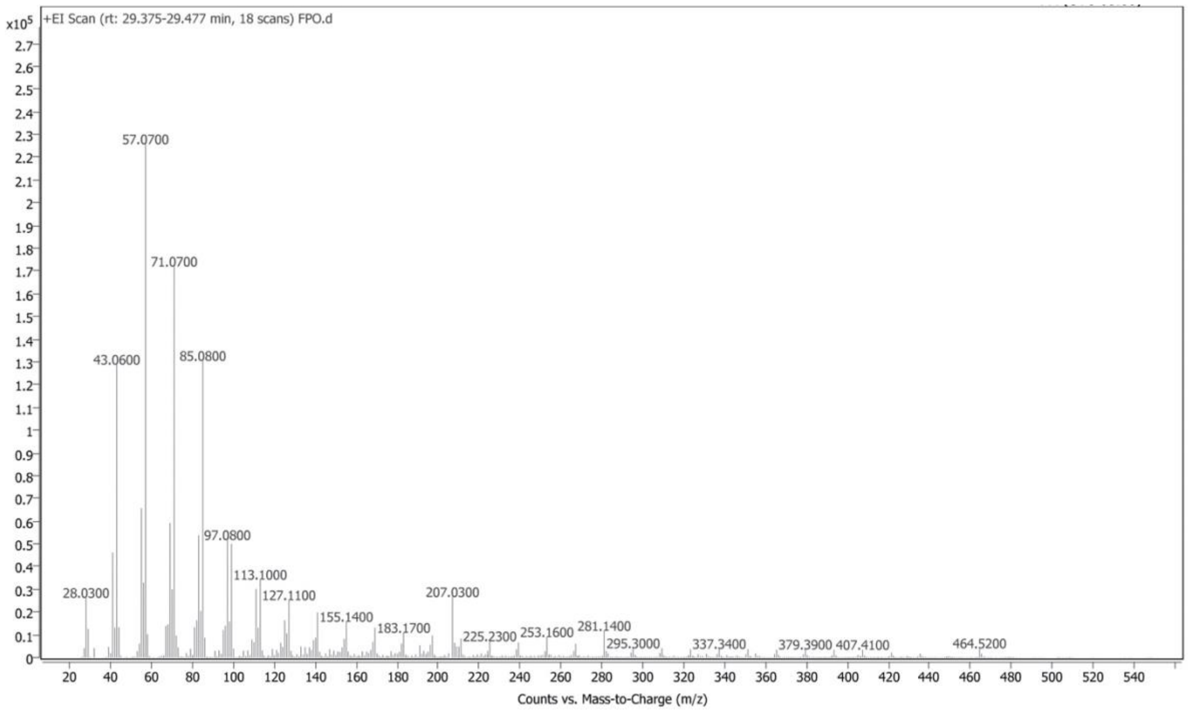

Peak 36

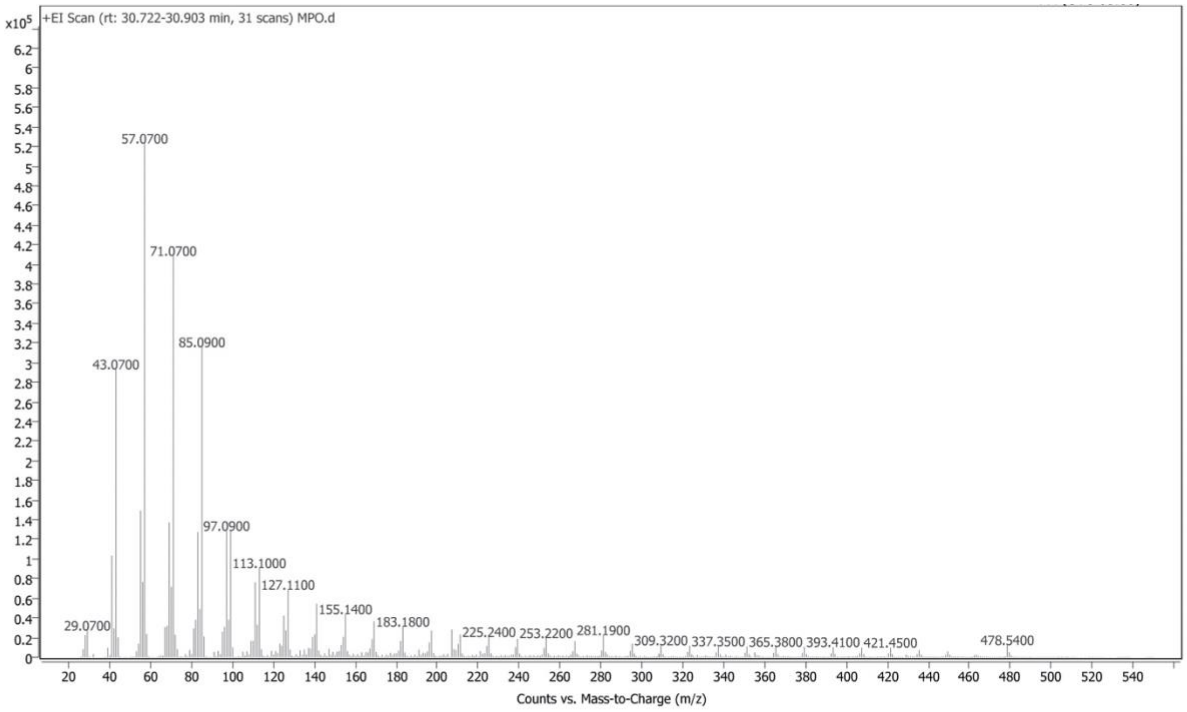
+EI Scan (rt: 30.722-30.903 min, 31 scans) MPO.d
x10^5
57.0700
71.0700
85.0900
43.0700
97.0900
113.1000
127.1100
155.1400
183.1800
29.0700
225.2400
253.2200
281.1900
309.3200
337.3500
365.3800
393.4100
421.4500
478.5400
Counts vs. Mass-to-Charge (m/z)

Peak 37
